# PARP-1/PARP-2 double deficiency in mouse T cells results in faulty immune responses and T lymphomas

**DOI:** 10.1038/srep41962

**Published:** 2017-02-09

**Authors:** Judith Navarro, Beatriz Gozalbo-López, Andrea C. Méndez, Françoise Dantzer, Valérie Schreiber, Carlos Martínez, David M. Arana, Jordi Farrés, Beatriz Revilla-Nuin, María F. Bueno, Coral Ampurdanés, Miguel A. Galindo-Campos, Philip A. Knobel, Sandra Segura-Bayona, Juan Martin-Caballero, Travis H. Stracker, Pedro Aparicio, Margarita Del Val, José Yélamos

**Affiliations:** 1 Cancer Research Program, Hospital del Mar Medical Research Institute (IMIM), Barcelona, Spain; 2 Inmunología Viral, Centro de Biología Molecular Severo Ochoa (CSIC-UAM), Madrid, Spain; 3 Biotechnology and Cell Signaling, UMR7242-CNRS, Laboratory of Excellence Medalis, ESBS, Illkirch, France; 4 Experimental Pathology Unit, IMIB-LAIB-Arrixaca, Murcia, Spain; 5 Centro de Investigación Biomédica en Red de Enfermedades Hepáticas y Digestivas, Madrid, Spain; 6 Genomic Unit. IMIB-LAIB-Arrixaca, Murcia, Spain; 7 Institute for Research in Biomedicine (IRB Barcelona), The Barcelona Institute of Science and Technology, Barcelona, Spain; 8 Barcelona Biomedical Research Park (PRBB), Barcelona, Spain; 9 Department of Biochemistry, Molecular Biology and Immunology, University of Murcia, Murcia, Spain; 10 Department of Immunology, Hospital del Mar, Barcelona, Spain

## Abstract

The maintenance of T-cell homeostasis must be tightly regulated. Here, we have identified a coordinated role of Poly(ADP-ribose) polymerase-1 (PARP-1) and PARP-2 in maintaining T-lymphocyte number and function. Mice bearing a T-cell specific deficiency of PARP-2 in a PARP-1-deficient background showed defective thymocyte maturation and diminished numbers of peripheral CD4^+^ and CD8^+^ T-cells. Meanwhile, peripheral T-cell number was not affected in single PARP-1 or PARP-2-deficient mice. T-cell lymphopenia was associated with dampened *in vivo* immune responses to synthetic T-dependent antigens and virus, increased DNA damage and T-cell death. Moreover, double-deficiency in PARP-1/PARP-2 in T-cells led to highly aggressive T-cell lymphomas with long latency. Our findings establish a coordinated role of PARP-1 and PARP-2 in T-cell homeostasis that might impact on the development of PARP-centred therapies.

T-cell development is initiated in the thymus from bone-marrow derived progenitors, giving rise to mature T-cells that will seed the peripheral lymphoid tissues^1^. Further development and differentiation continues in the periphery, and is critical for T-cells to attain full competence to provide appropriate immune responses to antigen challenge^2^. The balance between cell division and programmed cell death during T-cell development and differentiation must be tightly regulated to guarantee maintenance of T-cell homeostasis throughout life^2^. Two main environmental signals govern peripheral T-cell homeostasis: (i) the engagement of the antigen-specific T-cell receptor (TCR) by peptide presented on the major histocompatibility complex (MHC) molecules, and (ii) cytokine-mediated signals such as interleukin-7 (IL-7) and IL-15^2^,^3^. In addition to these environmental signals, cell intrinsic factors that modulate cell-cycle checkpoints, DNA repair processes and apoptosis must be integrated delicately in T-cells to maintain genomic stability and therefore contribute to the control of T-cell development and homeostasis^4^,^5^.

We report here a critical role of Poly(ADP-ribose) polymerase-1 (PARP-1) and PARP-2 in maintaining T-cell homeostasis and function. PARP-1 and PARP-2 belong to a family of enzymes that catalytically cleave β-NAD^+^ and transfer an ADP-ribose moiety onto residues of acceptor proteins, modifying their functional properties through poly(ADP-ribosyl)ation^6^,^7^. Defects in the maintenance of chromosome structure and DNA repair have been observed in mice upon deletion of either PARP-1 or PARP-2, supporting shared functions of PARP-1 and PARP-2 in maintaining genome integrity^8^.

Accordingly, PARP inhibitors have gained significant attention as new therapeutic drugs for cancer treatment^9^,^10^. However, PARP inhibitors currently in clinical trials or approved for clinical use^9^ are still unable to discriminate between individual PARP-isoforms, despite increasing biochemical and structural evidence that PARP family proteins play specific roles in the DNA-damage response and other cellular processes. Indeed, PARP-1 and PARP-2 can become selectively activated by specific stimuli, have different targets and/or interact with specific protein partners^7^,^11^,^12^,^13^,^14^, suggesting distinct biological functions that are beginning to be elucidated. Some of the biological processes in which PARP-2, but not PARP-1, have been specifically implicated are associated with cell types or processes that have high levels of proliferation, including spermatogenesis^15^, hematopoiesis under stress conditions^16^, erythropoiesis^17^, IgH/c-myc translocations during immunoglobulin class switch recombination^18^ and thymopoiesis^19^,^20^.

Although peripheral T-cell homeostasis seems to be normal in either PARP-1 or PARP-2-deficient mice^19^, several experimental data suggest a role of either PARP-1 or PARP-2 in T-cell biology. In addition, other PARPs, including PARP-14, have been implicated in T-cell mediated inflammation and gene regulation^21^. PARP-1 is involved in the regulation of nuclear factor of activated T-cells (NFAT)^22^, and forkhead box protein 3 (Foxp3)^23^,^24^. Moreover, PARP-1-deficiency biases T-cell responses to a Th1 phenotype^25^. While PARP-1 is dispensable for thymocyte development, PARP-2-deficiency produces a two-fold reduction in CD4^+^CD8^+^ double-positive (DP) thymocytes associated with decreased DP cell survival^19^. However, the effect of PARP-1 and PARP-2 double deficiency in T-cells remains unknown.

Here, to overcome the early lethality of PARP-1/PARP-2-double-mutant embryos^26^ and to clarify the specific and redundant functions of PARP-1 and PARP-2 in T-cell biology, we have generated and analysed PARP-1-deficient mice with a *Cd4*-promoter-driven conditional deletion of PARP-2 in T-cells. We show that simultaneous disruption of both proteins starting in DP thymocytes leads to impaired thymocyte maturation and loss of homeostasis in peripheral T-cells. Moreover, this alteration in peripheral T-cells compromises the immune response and results in the development of highly aggressive T-cell lymphomas with long latency. Our findings establish a redundant role of PARP-1 and PARP-2 in T-cell function that may have implications for the development of PARP-centred therapies.

## Results

### Generation of mice with a T-cell-specific deletion of PARP-2 in a PARP-1-deficient background

**Figure 1:**
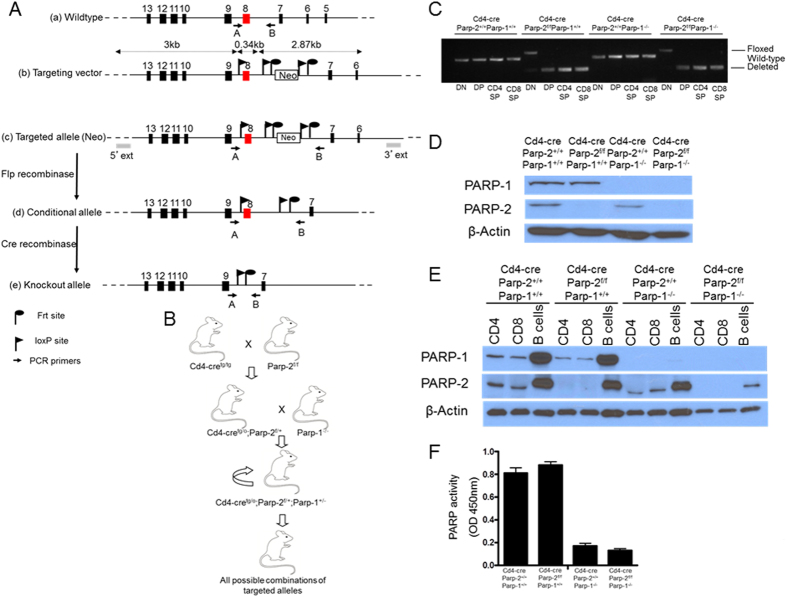
Generation of mice with a T-cell-specific deletion of PARP-2 in a PARP-1-deficient background. (**A**) Schematic representation of (a) the wild-type allele of the mouse *Parp-2* gene and the location of the genotyping primers A and B; (b, c) the structure of the correctly targeted allele with the introduced neomycin resistance cassette and loxP and FRT sites, and the locations of genotyping primers; (d) the conditional allele (flox) produced by Flp-enhanced recombinase-mediated recombination of FRT sites flanking Neo and the locations of genotyping primers; and (e) the deleted allele produced by cre recombination of loxP sites surrounding exon 8 and the locations of genotyping primers. (**B**) Schematic representation of the cross-breeding performed to generate mice with a T-cell-specific deletion of PARP-2 in a PARP-1-deficient background. *Parp-2* floxed (*Parp-2*^*f/f*^) mice were crossed with *Cd4-cre*-transgenic mice, producing heterozygous offspring that were then crossed with *Parp-1*^−/−^ mice. *Cd4-cre*^*tg/o*^*;Parp-2*^*f*/+^;*Parp-1*^+/−^ mice were subsequently intercrossed, producing all possible combinations of *Parp-2, Parp-1* and *Cd4-cre* targeted alleles. (**C**) PCR analysis from genomic DNA in thymic double-negative (DN: CD4^−^CD8^−^), double-positive (DP: CD4^+^CD8^+^), CD4SP (CD4^+^CD8^−^), and CD8SP (CD4^−^CD8^+^) sorted subsets from *Cd4-cre;Parp-2*^+/+^*;Parp-1*^+/+^, *Cd4-cre;Parp-2*^*f/f*^*;Parp-1*^+/+^, *Cd4-cre;Parp-2*^+/+^;*Parp-1*^−/−^, and *Cd4-cre;Parp-2*^*f/f*^*;Parp-1*^−/−^ mice. (**D**) Western-blot analysis of PARP-1 and PARP-2 protein expression in sorted DP thymocytes and (**E**) in sorted CD4^+^, CD8^+^, and B-cells from spleen. (F) PARP activity in protein extracts from spleen T-cells after *in vitro* activation with anti-CD3 + anti-CD28. Resting T-cells were isolated from spleen, cultured 14 h in the presence of anti-CD3 + anti-CD28 monoclonal antibodies, lysed and PARP activity determined in protein extracts. Results represent the mean ± SEM of a representative experiment from two independent experiments carried out in triplicate.

In order to study the functions of PARP-1 and PARP-2 in T-cells, we have generated mice homozygous for floxed *Parp-2 (Parp-2*^*f/f*^) that were crossed to *Cd4-cre* mice to induce a T-cell-specific recombination. The resulting *Cd4-cre;Parp-2*^*f/*+^ mice were crossed with *Parp-1*^−/−^ mice to generate heterozygous mice that were intercrossed to give all possible combinations of targeted alleles (Fig. 1A and B). To check the deletion efficiency of *Parp-2* in CD4-expressing cells, thymocyte populations were sorted and the presence of the floxed allele was analysed by PCR. Complete loss of the floxed allele was observed in CD4^+^CD8^+^ (DP), CD4^+^CD8^−^ (CD4SP), and CD4^−^CD8^+^ (CD8SP) thymocytes, but not in CD4^−^CD8^−^ (DN) cells from *Cd4-cre;Parp-2*^*f/f*^ mice (Fig. 1C). As predicted from the pattern of gene deletion, the expression of PARP-2 protein was abolished in DP thymocytes from *Cd4-cre;Parp-2*^*f/f*^ mice (Fig. 1D). We have also analysed PARP-1 and PARP-2 protein expression in sorted B-cells, CD4^+^ T-cells and CD8^+^ T-cells from spleen by western-blot. Data show complete and selective loss of PARP-2 in *Cd4-cre;Parp-2*^*f/f*^ T-cells but not in B-cells (Fig. 1E). Upon *in vitro* anti-CD3 plus anti-CD28 activation, PARP activity was not affected in splenic single PARP-2-deficient T-cells compared to control T-cells, while a strong reduction was observed in single PARP-1-deficient T-cells and in T-cells from *Cd4-cre;Parp-2*^*f/f*^*;Parp-1*^−/−^ mice (Fig. 1F). This result is compatible with the fact that global PARP activity in T-cells is mainly dependent on PARP-1^19^.

### T-cell-specific ablation of PARP-2 in a PARP-1-deficient background impairs T-cell homeostasis

**Figure 2:**
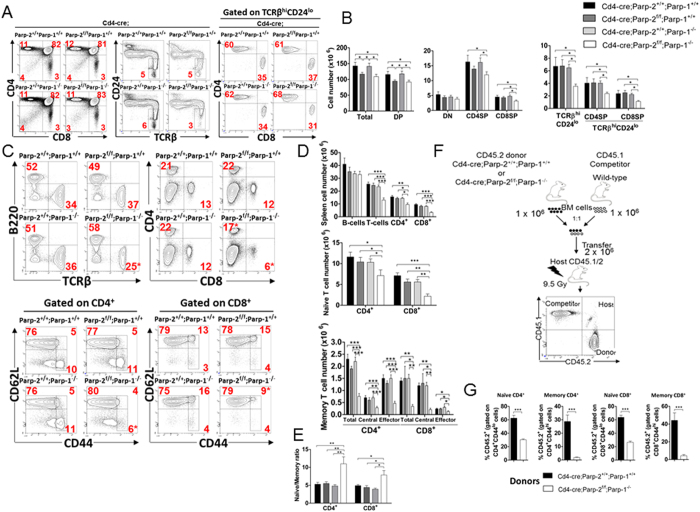
T-cell specific deletion of PARP-2 in a PARP-1-deficient background impairs T-cell homeostasis. (**A**) Representative dot-plots of CD4, CD8, TCRβ, and CD24 expression in thymocytes from 8 to 10-week-old mice of the indicated genotypes. Percentage of cells in the individual subpopulations is indicated in each quadrant. (**B**) Graph showing the absolute number of thymocytes in each population. (**C**) Representative dot-plots of TCRβ, B220, CD4, CD8, CD44 and CD62L expression in splenocytes of the indicated genotypes. Percentage of cells in the individual subpopulations is indicated in each quadrant. (**D**) Graph showing the absolute number of splenocytes in each population. Naïve, CD62L^+^ CD44^lo^; central memory, CD62L^+^ CD44^hi^; effector memory, CD62L^−^CD44^hi^. (**E**) Naïve/memory T-cell ratio in spleen. (**F**) Scheme describing the strategy for the generation of mixed bone marrow chimeras. Reconstituted cells were analyzed 10 weeks after transplantation. (**G**) Graph showing the frequency of CD45.2^+^ -expressing cells in spleen populations. Values represent the mean ± SEM of at least 8 mice of each genotype. *P < 0.05; **P < 0.01; ***P < 0.001.

We showed previously that depletion of PARP-2, but not PARP-1, in mice produced a reduction of DP thymocytes associated with a decreased DP survival, without affecting peripheral T-cell number^19^. To determine whether PARP-1-deficiency would influence the effects of PARP-2-deficiency on T-cell development, total thymocytes from PARP-1-single-mutant (*Cd4-cre;Parp-2*^+/+^;*Parp-1*^−/−^), PARP-2-single-mutant (*Cd4-cre;Parp-2*^*f/f*^*;Parp-1*^+/+^), PARP-1/PARP-2-double-mutants (*Cd4-cre;Parp-2*^*f/f*^*;Parp-1*^−/−^) and control (*Cd4-cre;Parp-2*^+/+^;*Parp-1*^+/+^) mice were counted, and thymocyte subsets were determined by flow cytometry (Fig. 2A and B). A decrease in total and DP number of thymocytes was observed in PARP-2-single-mutant and PARP-1/PARP-2-double-mutant T-cell mice compared to age-matched PARP-1-single-mutant or control mice (Fig. 2B), indicating that PARP-1 is dispensable during thymocyte development, in agreement with our previous data^19^. Further analysis of thymocytes revealed that only PARP-1/PARP-2-double-mutant mice exhibited a reduction in the number of SP mature thymocytes in both the CD4 and CD8 lineages (Fig. 2A and B), suggesting that PARP-1/PARP-2 doubly-deficient committed SP thymocytes have a maturation defect.

Consistent with a reduction of mature CD4SP and CD8SP thymocytes, T-cell-specific deletion of PARP-2 in a PARP-1-deficient background also resulted in a significant decrease in both the proportion and number of T-cells in the spleen, while no differences were observed in the B-cell compartment (Fig. 2C and D). Although both CD4^+^ and CD8^+^ T-cell proportion and number were decreased in the absence of both PARP-1 and PARP-2, the CD8^+^ T-cell population was affected more dramatically, as indicated by the CD4/CD8 ratio, which was 2-fold higher in the doubly deficient mice (ratio of 3.4) than in single-mutants (ratios of 1.9) or control mice (ratio of 1.7). The overall decreased T-cell number impacted the number of CD4^+^ and CD8^+^ naïve (CD62L^+^ CD44^lo^) cells, but a specific deficiency both in central memory (CD62L^+^ CD44^hi^) and in effector memory (CD62L^−^CD44^hi^) T-cells^27^ was also very markedly observed in mice with a double deficiency of PARP-1 and PARP-2 (Fig. 2C and D). The imbalanced ratio of naïve/memory T-cells in the spleen of PARP-1/PARP-2 doubly deficient mice points towards a decreased generation and/or survival of memory T-cells (Fig. 2E). Treg cells, defined as CD4^+^CD25^+^ FoxP3^+^, were slightly increased in PARP-1 single mutants compared to control mice, in agreement with previous reports^28^. However, this effect was not further exacerbated in the absence of PARP-2 (Figure S1).

To evaluate whether PARP-1/PARP-2-doubly-deficient T-cells had an intrinsic defect, we generated bone-marrow chimeric mice by reconstituting lethally irradiated mice (CD45.1/2) with a 1:1 mixture of CD45.1-competitor wild-type and CD45.2-control or PARP-1/PARP-2-double-mutants donor bone-marrow cells (Fig. 2F). After lethal irradiation, a significant number of T-cells are recipient-derived in agreement with previous studies^29^, and are enriched in CD44^hi^ memory cells, which are more resistant to γ-irradiation^30^. The contribution of CD45.2 PARP-1/PARP-2-double-mutant cells to all peripheral T-cell subsets was compromised, severely affecting memory T-cells (Fig. 2G and S2).

IL-7 is the major survival factor for mature thymocytes and peripheral naïve T-cells^31^,^32^. Accordingly, we examined IL-7Rα expression on mature thymocytes and peripheral T-cells. IL-7Rα expression on CD4SP and CD8SP thymocytes was similar in all genotypes (Figure S3A). In the spleen, doubly deficient naïve CD4^+^ and CD8^+^ T-cells expressed lower levels of IL-7Rα than control and single-deficient naïve CD4^+^ and CD8^+^ T-cells (Figure S3B). To determine the functional consequences of the slight decrease in IL7Rα expression on the ability to respond to IL-7, we examined whether recombinant IL-7 was able to support survival of T-cells *in vitro*. In the absence of IL-7, survival of thymocytes and peripheral T-cells was similar in all genotypes. Addition of IL-7 enhanced survival of cells from all genotypes to similar levels (Figures S3C and S3D), suggesting that IL-7 survival response is unaltered in PARP-1/PARP-2 doubly deficient T-cells. Our results agree with previous data showing that small differences in IL-7Rα expression are not necessarily biologically significant^33^, consistent with the fact that IL-7Rα heterozygous mice do not have a peripheral T-cell defect^34^,^35^.

Altogether, our data indicate a compensatory or redundant function of PARP-1 and PARP-2 in T-cell homeostasis resulting in a profound and selective alteration in the T-cell compartments when both proteins are missing.

### T-cell-specific disruption of PARP-2 in a PARP-1-deficient background leads to DNA damage and T-cell death

**Figure 3:**
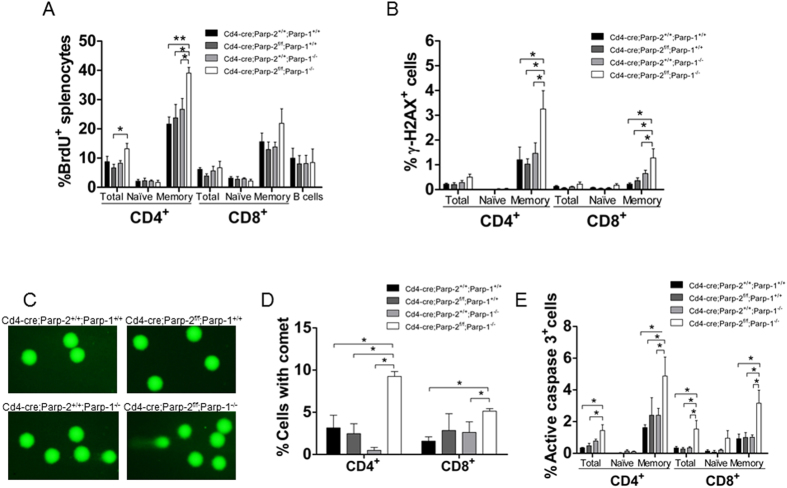
Effect of PARP-1/PARP-2 double deficiency on T-cell proliferation, DNA damage and apoptosis. (**A**) *In vivo* proliferation of T-cell subsets. Eight-to-ten-week-old mice were i.p. injected with BrdU at 24 and 12 h before sacrifice. Cell suspensions from spleen were stained with anti-CD4, anti-CD8, anti-CD62L, anti-CD44 and anti-B220 to identify cell subsets, with anti-BrdU to identify cells synthesizing DNA and with DAPI to stain for the total DNA content in the cells. BrdU incorporation in each population was analysed by flow cytometry. Bars represent the mean ± SEM values of the percentage of BrdU^+^ cells. (**B**) Graph showing the mean ± SEM values of the percentage of γH2AX^+^ cells in each T-cell subset, determined by flow cytometry. (**C**) Representative image showing DNA damage in CD4^+^ splenic T-cells derived from mice of the indicated genotypes, visualized by the alkaline comet assay. (**D**) Graph showing the percentage of T-cells with comet. An average of 100 cells was scored from each mouse. (**E**) Graph showing the percentage of active caspase-3-positive T-cell in each subsets, determined by flow cytometry. Bars represent the mean ± SEM values obtained from at least 6 mice per genotype from two independent experiments. *P < 0.05; **P < 0.01; ***P < 0.001.

To evaluate whether the reduced peripheral T-cell number in mice bearing a double deficiency of PARP-1 and PARP-2 was secondary to proliferation defects, we measured the proportion of T-cells that entered S-phase using BrdU incorporation over a period of 24 hours *in vivo*. The percentage of BrdU^+^ cells was increased in spleen CD4^+^ memory T-cells from mice doubly deficient for PARP-1 and PARP-2 compared to control and either PARP-1 or PARP-2 single mutants. A slight increase in the percentage of BrdU^+^ cells in CD8^+^ memory T-cells from mice doubly deficient for PARP-1 and PARP-2 was also observed, although differences were not statistically significant, while a similar percentage of cells incorporating BrdU were observed in naïve T-cells, and B-cells from the four genotypes (Fig. 3A). Our data are consistent with doubly deficient T-cells having no defect in initiating DNA synthesis in response to a proliferation stimulus, likely in order to replenish the partially void memory T-cell niche. The reduced number of cells in this niche could be the result of increased cell death as a consequence of elevated DNA-strand breaks, rather than compromised homeostatic proliferation.

To address this, we used flow cytometry to evaluate the phosphorylation status of histone H2AX (γ-H2AX). An increased percentage of γ-H2AX^+^ - cells was detected in doubly-deficient memory T-cells, while no differences were observed in naïve T-cells (Fig. 3B). We next performed an alkaline comet assay to evaluate induction and repair of DNA breaks independently of their signaling and processing markers. We detected a significant increase in the number of cells exhibiting detectable DNA damage, inferred from the comet shape in PARP-1/PARP-2 doubly deficient T-cells from both CD4 and CD8 lineages, compared to control and single mutant T-cells (Fig. 3C and D). In agreement with this, we observed significantly higher numbers of apoptotic cells, identified by staining for cleaved caspase-3, in PARP-1/PARP-2-doubly-deficient memory T-cells than in control and single mutant T-cells (Fig. 3E). Together, our data indicate that the loss of both PARP-1 and PARP-2 result in spontaneous DNA damage and elevated apoptosis, resulting in T-cell lymphopenia.

### Mice doubly deficient for PARP-1 and PARP-2 in T-cells exhibit a defective T-dependent antibody response

**Figure 4:**
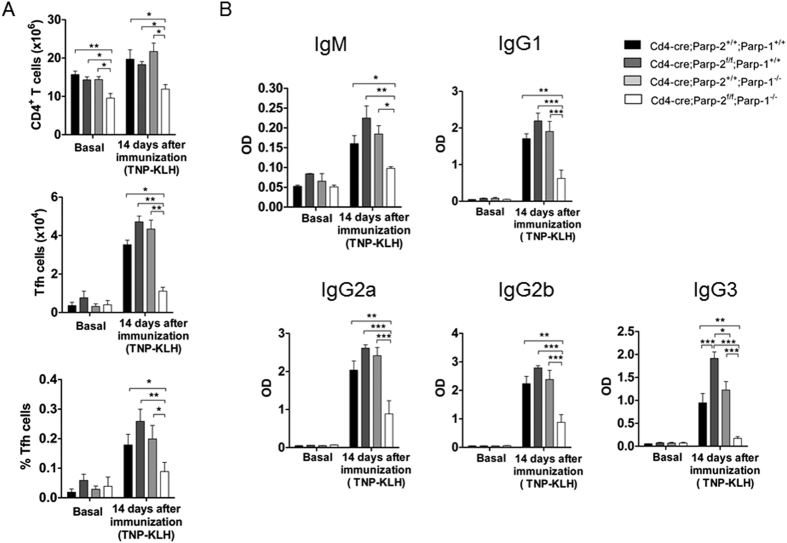
Mice carrying a T-cell specific deletion of PARP-2 in a PARP-1-deficient background display defects in specific antibody response to T-dependent antigens. Ten-to-twelve-week-old *Cd4-cre;Parp-2*^+/+^*;Parp-1*^+/+^, *Cd4-cre;Parp-2*^*f/f*^*;Parp-1*^+/+^, *Cd4-cre;Parp-2*^+/+^;*Parp-1*^−/−^, and *Cd4-cre;Parp-2*^*f/f*^*;Parp-1*^−/−^ mice were i.p. injected with the T-dependent antigen TNP-KLH and sigma-system adjuvant. (**A**) T_fh_ cell development is impaired in *Cd4-cre*;*Parp-2*^*f/f*^;*Parp-1*^−/−^ mice. Spleen samples from no-immunized mice and fourteen days after immunization, were collected and cells were counted and stained to detect by flow cytometry total CD4^+^ T-cells and T_fh_ cells (CD4^+^CXCR5^+^ PD1^+^ ICOS^+^). Graph showing the number of CD4^+^ and number and percentage of T_fh_ cells. (**B**) Serum was collected at the indicated time point, and TNP-specific IgM, IgG1, IgG2a, IgG2b and IgG3 were assayed by ELISA. Bars represent the mean ± SEM values of at least 7 mice of each genotype. *P < 0.05; **P < 0.01; ***P < 0.001.

To determine whether PARP-1 and/or PARP-2-deficiency in T-cells compromised the *in vivo* responsiveness to specific T-dependent antigenic challenge, mice were immunized with a T-dependent antigen (TNP-KLH) and cells from the spleen were analysed at day 14 post-immunization. Similarly to unimmunized mice, the number of CD4^+^ T-cells was significantly decreased in immunized mice lacking both PARP-1 and PARP-2 in their T-cells, compared to control and single mutant mice (Fig. 4A). The CD4^+^ -T-follicular helper cells (T_fh_) are the true helper cells for antibody responses^36^. Mice from the four genotypes exhibited low numbers of T_fh_ cells, defined as CD3^+^CD4^+^CXCR5^+^PD1^+^ICOS^+^, at baseline. At day 14 post-immunization, both the percentages and the absolute numbers of T_fh_ cells increased in control and single mutant mice. However, T_fh_ expansion was strikingly impaired in mice with PARP-1/PARP-2 doubly deficient T-cells (Fig. 4A). Accordingly, TNP-specific IgM, IgG1, IgG2a, IgG2b, and IgG3 levels at day 14 post-immunization were significantly reduced in mice bearing a double deficiency of PARP-1 and PARP-2 in T-cells compared with control and single mutants (Fig. 4B). This defect is most likely due to a defect in T help, as the intrinsic ability of B cells from *Cd4-cre;Parp-2*^*f/f*^*;Parp-1*^−/−^ mice to undergo class switch recombination (CSR) *ex vivo* was similar to B cells from control mice and single PARP-1 deficient mice^18^ (Figure S4).

### T-cell-specific ablation of PARP-2 in a PARP-1-deficient background impairs the primary cellular and humoral immune responses to vaccinia virus infection

**Figure 5:**
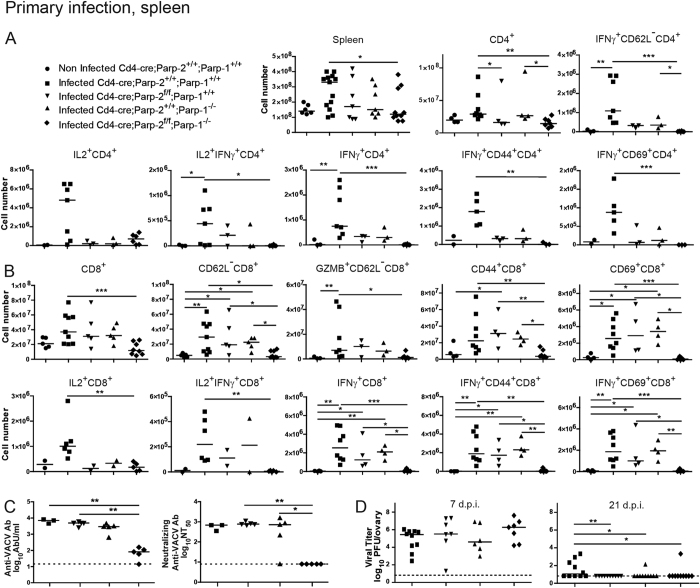
Primary cellular and humoral immune response in splenocytes following vaccinia virus infection. Mice of the indicated genotypes were infected with VACV-flOVA and 7-9 days later spleens were extracted. (**A**) Flow cytometry analysis of activation molecules (CD44, CD69, CD62L) and effector molecules (IL2, IFNγ) (alone or combined) in the CD4^+^ cell population following *ex vivo* incubation with VACV-infected dendritic cells. (**B**) Analysis of activation molecules (CD44, CD69, CD62L) and effector molecules (GZMB, IL2, IFNγ) (alone or combined) in CD8^+^ cells following *ex vivo* incubation with B8R_20-27_ peptide. (**C**) Mice were infected with VACV-flOVA and 21 days later serum was extracted and inactivated. Antibodies anti-VACV (left panel) were determined using ELISA and titers were expressed in antibody units (AbU)/mL. Neutralizing antibodies anti-VACV (right panel) were assessed by plaque assay and the neutralizing titer 50% (NT_50_) was determined. Detection limit is indicated by the dashed line. Dots represent individual mice and horizontal lines represent median values of cell numbers or antibody titers for each genotype. (**D**) VACV replication *in vivo*. Mice were infected with VACV-flOVA and 7 or 21 days later ovaries were extracted and virus titrated. Dots represent individual ovary titers and horizontal lines represent median values. Detection limit is indicated by the dashed line. *P < 0.05; **P < 0.01; ***P < 0.001.

We next studied the immune response to vaccinia virus (VACV) infection. Mice of the four genotypes studied were infected with VACV-flOVA expressing ovalbumin and the primary antiviral cellular immune response was analyzed 1 week later. Compared with control and single PARP-1 or PARP-2-deficient mice, major differences were found in PARP-1/PARP-2-double-mutant mice, which had fewer splenocytes and also lower numbers of CD4^+^ cells (Fig. 5A and S5A) and CD8^+^ cells (Fig. 5B and S5B). Co-culture with VACV-flOVA infected dendritic cells (DC) showed a defective primary response of CD4^+^ T-cells in *Cd4-cre;Parp2*^*f/f*^*;Parp1*^−/−^ mice. The number and percentage of CD4^+^ T-cells secreting IL-2 and specially IFNγ was lower in these mice, especially when considering activated cells (CD62L^−^, CD44^+^ or CD69^+^) that secreted IFNγ. Some decrease in single PARP-1*-* and single PARP-2-deficient mice was also appreciated (Fig. 5A and S5A). The fact that the defects are additive suggests that PARP-1 and PARP-2 play partially redundant roles in T lymphocyte antiviral function. Indeed, PARP-1/PARP-2-doubly-deficient CD4^+^ T-cells had a defect in the production of IFNγ and IL-4 *in vitro* (Figure S6).

Regarding CD8^+^ cells (Fig. 5B), the granzyme B (GZMB)^+^ CD62L^−^ population was also analysed, as it has been identified to include most T-lymphocytes activated in response to VACV^37^ in contrast to GZMB^−^CD62L^+^ -cells, which identify naïve lymphocytes. This population, directly measured *ex vivo*, was diminished in *Cd4-cre;Parp2*^*f/f*^*;Parp1*^−/−^ mice, indicating a defect in the virus-specific cytotoxic response. A marked defect in activation was also detected as shown by the decreased numbers of doubly deficient activated CD8^+^ T-lymphocytes (CD62L^−^, CD44^+^, and specially CD69^+^) cells. In addition, after culturing splenocytes for 6 h with VACV immunodominant B8R_20-27_ peptide, fewer cells from *Cd4-cre;Parp2*^*f/f*^*;Parp1*^−/−^ mice secreted IL-2 and IFNγ. The defect in IFNγ production was even more severe in activated cells (CD44^+^ or CD69^+^). Viral specificity was assured throughout by subtracting background in the absence of infection of dendritic cells or of peptide antigen. As for CD4^+^ T lymphocytes, a decrease in CD8^+^ T lymphocyte populations was also detected in single PARP-1- and single PARP-2-deficient mice (Fig. 5B). Supporting results were found in peritoneal exudate cells from the same infected mice (Figure S7).

To analyze the primary humoral response to VACV infection, antibody production was quantified by ELISA in serum extracted 21 days post infection. (dpi) (Fig. 5C). PARP-1/PARP-2-double-mutant mice had a 100-fold impaired anti-VACV antibody response compared to single mutants or control animals. Anti-VACV neutralizing antibodies were also measured by virus plaque reduction assay, detecting also a similarly strong impairment in PARP-1/PARP-2-double-mutant mice (Fig. 5C). Altogether, the data suggest that PARP-1 and PARP-2 proteins are necessary for generating virus-specific cellular and humoral responses to VACV.

Ovary viral titers were determined at 7 dpi, showing that the virus replicated similarly in the four genotypes. This indicates that all genotypes were equally infected (Fig. 5D). At 21 dpi mice of all genotypes were surprisingly able to clear the virus (Fig. 5D) suggesting that the overall immune response in all of the PARP-deficient backgrounds was sufficient to fight infection.

Bone-marrow derived DC from each genotype were also generated. While a minor improvement was detected in differentiation to DCs, no differences were found in maturation, susceptibility to VACV infection or in the capacity of VACV-infected BMDCs to prime naïve OT-I CD8^+^ T-cells (Figure S8). These data support the notion that the *in vivo* defect is restricted to lymphocytes and not to the antigen presenting cells. While the DC from the *Cd4-cre;Parp-2*^*f/f*^*;Parp-1*^−/−^ animals should retain PARP-2 expression as DC from the *Cd4-cre;Parp2*^+/+^*;Parp1*^−/−^, it is noteworthy that DC obtained from fully deficient PARP-2 mice also showed no defect (Figure S8). It cannot be excluded that potential deficiencies in CD4^+^ DC in *Parp-2*^*f/f*^ animals may contribute to the profound lymphocyte defect in *Cd4-cre;Parp-2*^*f/f*^*;Parp-1*^−/−^, but it is unlikely, as the lymphocyte defect already showed a tendency towards faulty antiviral function in animals singly deficient for either *Parp* gene (Fig. 5).

### T-cell-specific disruption of PARP-2 in a PARP-1-deficient background impairs CD8^+^ T-lymphocyte-mediated vaccination to vaccinia virus infection

**Figure 6:**
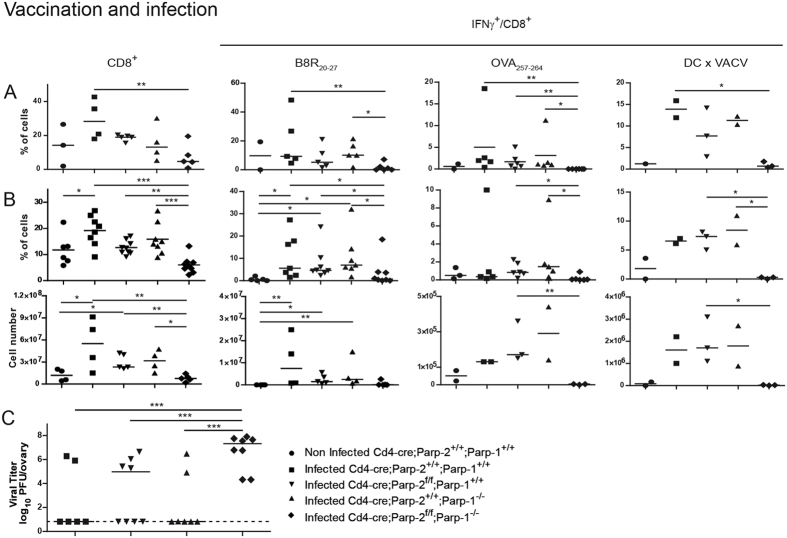
Secondary cellular immune response following vaccinia virus infection of vaccinated animals. Mice were vaccinated with BMDCs loaded with MHC class I-restricted B8R_20-27_ and OVA_257-264_ peptides and infected 9-21 days later with VACV-flOVA. Peritoneal cells (**A**) and splenocytes (**B**) were extracted 5 days later and cells were incubated *ex vivo* with B8R_20-27_ or OVA_257-264_ peptides or with VACV-flOVA-infected DC, as indicated. IFNγ production was measured in CD8^+^ cells by flow cytometry. Dots represent individual mice and horizontal lines represent median values for each genotype. (**C**) Ovaries were extracted and virus titrated. Dots represent mean ovary virus titres of each animal and horizontal lines represent median values for each genotype. Detection limit is indicated by the dashed line. *P < 0.05; **P < 0.01; ***P < 0.001.

In order to study the impact of the PARP deficiencies in the function of memory T-lymphocytes, we tested the hallmark of memory, which is to protect through vaccination against a subsequent infection. In order to induce separately the CD8^+^ T-lymphocyte response in a model with no contribution from a virus-specific CD4^+^ T-cell response, mice were vaccinated with DC pulsed with B8R and OVA peptides presented by MHC class I molecules, and then infected with VACV. Following this vaccination protocol, virus replication in wild-type mice is strongly reduced and most mice fully control it^38^. At day 5 pi the secondary CD8^+^ T-lymphocytes were co-cultured with B8R and OVA peptides or with VACV-flOVA-infected DC presenting all possible vaccinia antigens. As in the primary response, there were fewer secondary CD8^+^ T-cells and they failed to mount a proper IFNγ response in *Cd4-cre;Parp2*^*f/f*^*;Parp1*^−/−^ mice (Fig. 6). This defect strongly impacted virus control, to the point that vaccination had little effect in the double deficient background (Fig. 6C). Interestingly, there was an inverse correlation in doubly deficient animals, in that all mice with high virus titres above 6.8 log units in Fig. 6C had less than 2% IFNγ^+^/CD8^+^ cells in Fig. 6A,B (correlation not shown). These results indicate that the CD8^+^ T lymphocyte response alone is not able to control VACV infection *in vivo* in doubly deficient animals, but the correlation shows that it probably contributes to it. Since primary virus infection was controlled by all genotypes (Fig. 5D), this suggests the possibility that the combination of a reduced CD8^+^ T-cell response with impaired CD4^+^ T-cells and antibody production during the acute response may explain virus control after primary infection even in the *Cd4-cre;Parp2*^*f/f*^*;Parp1*^−/−^ mice, as these three immune responses are each relevant to control VACV acute infection^39^,^40^.

### PARP-1 and PARP-2 play redundant roles in tumour suppression in T-cells

**Figure 7:**
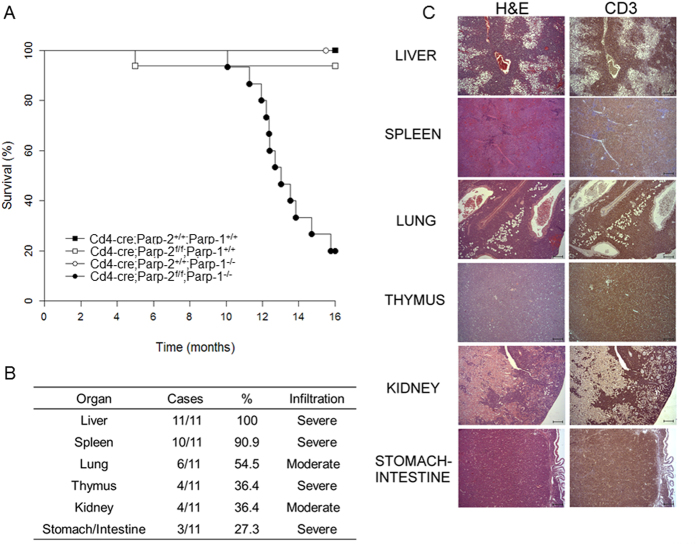
T-cell specific deletion of PARP-2 in a PARP-1-deficient background leads to death of mice with T-cell lymphomas. (**A**) Kaplan-Meier survival curves for *Cd4-cre;Parp-2*^+/+^*;Parp-1*^+/+^(n = 9), *Cd4-cre;Parp-2*^*f/f*^*;Parp-1*^+/+^(n = 16), *Cd4-cre*;*Parp-2*^+/+^;*Parp-1*^−/−^ (n = 8), and *Cd4-cre;Parp-2*^*f/f*^*;Parp-1*^−/−^ (n = 15) mice. Percent survival is plotted as a function of time in months. The difference in survival between *Cd4-cre;Parp-2*^*f/f*^*;Parp-1*^−/−^ and the other three genotypes was highly significant (p < 0.001) by log-rank test. (**B**) Summary of organs with highly invasive T-cell lymphoma cells. For evaluation of the infiltrative degree of tumors, a semi-quantitative scale comprising 4 grades was used: 0 (no infiltration), 1 (mild infiltration), 2 (moderate infiltration) and 3 (severe infiltration). The overall infiltrative value of each organ was established by calculation of the mean of all values obtained. (**C**) Hematoxylin and eosin (left panel) and anti-CD3 (right panel) staining of fixed tissue sections reveal that mortality of *Cd4-cre;Parp-2*^*f/f*^;*Parp-1*^−/−^ mice is due to T-cell lymphomas. Scale bar: 0.2 mm.

Interestingly, mice with PARP-1/PARP-2-double-mutant T-cells started to die spontaneously at the age of 10 months and about 80% had died by 16 months (Fig. 7A). On the contrary, no mortality was observed in single mutant or control mice during this time period. The main pathologic feature identified in these animals was the presence of a highly invasive cellular population in several organs (Fig. 7B) with round and euchromatic nuclei with well-developed nucleoli and basophilic cytoplasm, and a high mitotic index (>10 mitoses/high power field). Immunohistopathological analysis identified these neoplastic lesions as T-cell lymphomas, determined by CD3 expression (Fig. 7C). The main affected organs were the liver, spleen, lungs, kidney, thymus, and intestinal tract (Fig. 7B and C). Based on the severe damage that affected several vital organs, mainly the liver, the progression of the neoplasia in such organs could be correlated with, and was the likely cause of death in these animals.

## Discussion

Maintenance of T-cell number during homeostasis and upon antigen challenge must be tightly regulated to provide appropriate immune responses and prevent immunopathology. In addition to MHC-TCR interaction and cytokine-mediated signals, cell intrinsic factors that modulate essential functions of T-cells, are critical to preserve genomic stability and contribute to normal T-cell development^4^,^5^. Previously, we have demonstrated a role for the DNA damage response-associated protein PARP-2, but not PARP-1, in thymocyte development, without affecting peripheral T-cell homeostasis^19^,^20^. Here, we have further elucidated redundant and non-redundant functions of PARP-2 and PARP-1 in T-cells, using conditional deletion of PARP-2 in the T-cell lineage to overcome the lethality of PARP-1/PARP-2 doubly deficient embryos^26^.

Our results indicate that double deficiency of PARP-1 and PARP-2 impacts all T-cell compartments. In addition to the reduction of DP thymocytes associated with the loss of PARP-2^19^, the double deficiency of PARP-1/PARP-2 in T-cells affects CD4SP and CD8SP T-cell maturation in the thymus, and leads to a strong reduction of both naïve and memory T-cells in the periphery, despite a normal response to IL-7, the major T-cell survival cytokine for mature thymocytes and naïve and memory T-cells^31^,^32^. T-cell lymphopenia can be explained by a shift in the balance between proliferation and survival during T-cell homeostatic expansion to replenish the niche^41^. Interestingly, our *in vivo* data revealed no defect in entering the S-phase after proliferation stimulus in peripheral T-cells from PARP-1/PARP-2 doubly deficient mice, but rather increased cell death. In agreement with a role for PARP-1 and PARP-2 in maintaining genomic stability^6^, we provide evidence that PARP-1 and PARP-2 are working in a redundant manner in proliferating T-cells to prevent the accumulation of toxic levels of unrepaired DNA damage that results in cell death upon homeostatic proliferation or in response to antigen challenge, but not in resting conditions. PARP-1 and PARP-2 maybe acting through the principle of synthetic lethality, which describes the process by which defects in two different genes together result in cell death but independently do not affect viability^42^. The stronger effect on the CD8^+^ T-cell population compared to the CD4^+^ subset in PARP-1/PARP-2 doubly deficient mice could be due to the higher susceptibility of MHC-class I-restricted thymocytes to apoptosis^43^ and to the faster proliferation of CD8^+^ than CD4^+^ T-cells^44^,^45^. In addition, bone-marrow transplantation experiments revealed a dramatic effect on the contribution of doubly-eficient PARP-1 and PARP-2 T-cells to the T-cell memory compartment, whose generation requires proliferation of naïve T-cells^46^. This redundant role for PARP-1 and PARP-2 in preserving T-cell homeostasis beyond the DP stage contrasts with other biological processes associated with a high proliferative cell rate, such as spermatogenesis^15^, stress induced hematopoiesis^16^ or erythropoiesis^17^, in which only PARP-2 appears to be specifically involved.

Notably, peripheral T-cell lymphopenia in PARP-1/PARP-2 doubly deficient mice results in functional impairment, as revealed by a dramatic defect in the humoral response to the T-dependent antigen TNP-KLH. Furthermore, this impairment was also observed with the infection with VACV. Virus infection also allowed testing of antibody function *in vivo*, demonstrating a remarkable 100-fold decrease in the virus-neutralizing titers. Overall, the impairment of antibody production can be accounted for by the dramatically decreased number of T_fh_ cells^36^ in *Cd4-cre;Parp-2*^f/f^;*Parp1*^−/−^ mice. Interestingly, treatment of mice with the PARP-1/PARP-2 inhibitor olaparib, blocks IgE production and prevents OVA challenge-induced elevation of CD4^+^ T-cells in a mouse model of asthma^47^.

In addition to T-cell lymphopenia affecting both the CD4^+^ and CD8^+^ compartment, virus infection also revealed a redundant role of PARP-1 and PARP-2 in T-cell effector function. Since PARP-1 deficiency alone did not lead to defects in IFNγ production in a setting without infection^48^, the effect must be ascribed either to the combined deficiency of both *Parp* genes or to the additional stress imposed by a virus that replicates extensively in the infected mice, which we report here for the first time. Lack of both *Parp* genes seemed to affect more severely CD8^+^ cell number and function. In addition, a separate analysis of the CD8^+^ T-cell function after vaccination in the absence of virus-specific CD4^+^ T-cell help showed that the secondary antiviral CD8^+^ response also had a profound deficit that compromised vaccination efficacy.

Although potential defects cannot be excluded in the repertoire of B8R-specific CD8^+^ T-lymphocytes in *Cd4-cre;Parp2*^*f/f*^*;Parp1*^−/−^ mice, this is unlikely, as B8R is the immunodominant VACV epitope in C57BL/6 background, and B8R-specific CD8^+^ T-lymphocytes are very polyclonal^49^. This issue does not apply to the CD4^+^ T-lymphocyte response, as they were stimulated *ex vivo* with infected DC displaying all possible epitopes, due to the lack of a clearly immunodominant MHC class II presented viral peptide. The B8R-specific TCR clonotypes seem to slightly vary from the primary to the memory phase^49^, which may account for the more extreme defect detected in the secondary CD8^+^ T-cell response to vaccination and infection, when compared with the acute phase of infection. Nevertheless, the fact that there is also a clear tendency for a defect when OVA peptide is used as a read-out makes unlikely the possibility that a defective TCR repertoire explains the deficiency observed in CD8^+^ T-lymphocytes, and rather suggests a purely functional impairment related mainly to IFNγ production by activated T-cells of both compartments.

**Figure 8:**
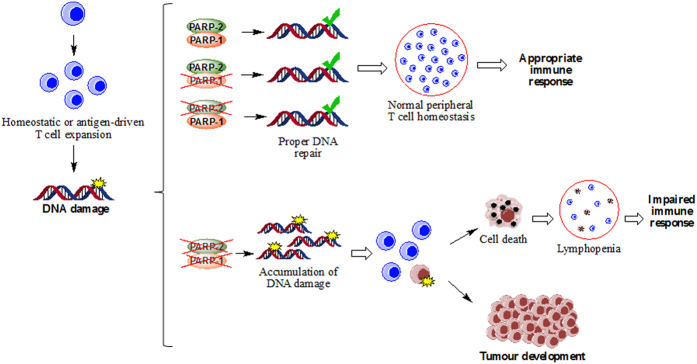
Model for T-cell lymphopenia, impaired immune response and development of T-cell lymphomas in mice harbouring a double deficiency of PARP-1 and PARP-2 in T-cells. We propose that DNA damage that arises following DNA replication during T-cell expansion can be repaired appropriately in cells lacking either PARP-1 or PARP-2. However, in doubly deficient T-cells, damage accumulates leading to cell death, lymphopenia and an impaired immune response, or to genetic lesions that can promote increased tumor development.

Altogether, our results are consistent with a model (Fig. 8) whereby double deficiency of PARP-1/PARP-2 in T-cells leads to DNA damage accumulation in cells intending to proliferate to replenish the memory niche during homeostasis and when proliferating in response to activation by antigen challenge, such as a virus infection. The presence of either PARP-1 or PARP-2 is sufficient to maintain genome integrity and prevent DNA-damage accumulation and apoptosis. These two processes are altered in PARP-1/PARP-2-doubly-deficient proliferating lymphocytes leading to lymphopenia and impaired immune responses. In addition, doubly deficient T-lymphocytes with an aberrant DNA-damage response occasionally survive with accumulated genetic abnormalities, resulting in T-cell lymphomas later in life. During T-cell clonal expansion, PARP-1 and PARP-2 may compensate for each other to maintain genomic stability. This assumption is sustained by our results and in agreement with previous data showing high levels of genomic instability leading to the embryonic lethality of *Parp-1*^−/−^*/Parp-2*^−/−^ mice^26^. Indeed, genomic instability in T-cells is associated with a preleukemic state^50^ and impairments in the DNA damage response in T-cells have been associated with the development of peripheral T-cell lymphomas^50^,^51^,^52^,^53^.

Currently there is considerable enthusiasm about the prospect of anti-cancer compounds that act through targeting PARP proteins, particularly in combination with defects in DNA damage signalling^54^,^55^,^56^,^57^. However, while non-isoform-selective PARP inhibitors are available, the current compounds lack the desired selectivity and may result in differential off-target effects. Although treatment with PARP inhibitors can never reach the level or the persistence achieved through the genetic knockout of PARP-1/PARP-2 in lymphocytes, our data demonstrating a redundant role for PARP-1 and PARP-2 in the T-cell immune response and tumour suppression in T-cells may have implications for the use of non-selective inhibitors in human patients. Importantly, our results highlight the importance of understanding the specific involvement of PARP-1, PARP-2, and other PARP family members in key biological processes that will provide a basis for the development and rational exploitation of PARP inhibitors.

## Methods

### Mice

The *Parp-2*^*flox/flox*^ (*Parp-2*^*f/f*^) mouse was established at the MCI/ICS (Institut Clinique de la Souris-ICS-MCI, Phenomin, Illkirch, France) as indicated in the supplementary data. *Parp-1*^−/−^ and transgenic mice for cre-recombinase driven by the *Cd4* promoter (*Cd4-cre*) have been described^58^,^59^. Genotyping was performed by PCR analysis using tail DNA and the primers listed in Table S1. All mice were kept under pathogen-free conditions and studies were approved by the PRBB and CSIC Animal Care Committees. All experiments were performed in accordance with relevant guidelines and regulations. *Parp-2* deletion on genomic DNA was confirmed by PCR analysis on FACS-purified thymocyte populations.

### Flow cytometry and cell sorting

Cell suspensions were washed in PBS, resuspended in PBS containing 0.5% BSA and incubated with antibodies on ice for 30 min. For intracellular staining, cells were stained for cell surface markers, fixed, and made permeable by using an intracellular staining buffer set (BD Biosciences), and then stained with specific antibody or isotype control. All antibodies used are indicated in the supplementary data. Annexin-V analysis was performed using an annexin-V apoptosis detection kit (BD Biosciences). Samples were acquired with FACS Calibur, FACS Canto or LSRFortessa cytometers and cell sorting was performed in a FACS AriaIISORP (BD Biosciences). Cell doublets were excluded from all analyses and dead cells were excluded by the use of DAPI. Data were analysed with the DIVA (BD Biosciences) and FlowJo (TreeStar) softwares.

### Cell isolation and culture

Cell suspensions from thymus and spleen were prepared and red blood cells from spleen were lysed using ACK lysis buffer (BioWhitaker). Cells resuspended in DMEM medium supplemented with 10% fetal bovine serum (FBS) were seeded in 96-well plates (1 × 10^5^ cells/well). When indicated, cells were treated with 10 ng/ml recombinant mouse IL-7 (Peprotech). Total T-cells were isolated from spleens of 8–12-week-old mice by magnetic depletion of non-T-cells using a MACS isolation kit, according to the manufacturer’s instructions (Miltenyi Biotec). Purity was assessed by flow cytometry analysis and all preparations were more than 98% pure. Untouched purified cells were resuspended in DMEM media supplemented with 10% FBS, seeded in 24-well plates (1 × 10^6^ cells/well) coated with anti-CD3 (5 μg/ml; BD Biosciences, clone 145-2C11), and anti-CD28 (5 μg/ml; BD Biosciences, clone 37.51) monoclonal antibodies. In order to determine CD8^+^ T-cell activation in *ex vivo* assays, splenocytes were stimulated with 10^−6^ M B8R_20-27_ or OVA_257-264_ peptides for 2 h, or with DC previously infected with VACV-flOVA for 5 h and then for 4 more hours in the presence of brefeldin A in αMEM medium supplemented with 5 × 10^−5^ M β-mercaptoethanol, 100 U/ml penicillin, 100 U/ml streptomycin, 10 μg/ml kanamycin, 2.5 μg/ml amphotericin and 10% FBS. When determining CD4^+^ T-cell activation, splenocytes were incubated with overnight VACV infected DCs for 2 h and 6 more hours in the presence of brefeldin A.

For CSR assays, B-cells were isolated from spleens of 8–12-week-old mice using CD43-microbeads (Miltenyi Biotec) and cultured for 3 days with LPS (50 μg/ml; Sigma-Aldrich) to switch to IgG2b and IgG3, LPS and IL-4 (5 ng/ml; Peprotech) to switch to IgG1 and LPS and IFNγ (100 ng/ml; Peprotech) to switch to IgG2a. CSR was assessed by flow cytometry. To measure T cell differentiation *in vitro*, naïve CD4^+^ T-cells were isolated from spleen, using a naïve CD4^+^ T-cell isolation kit according to the manufacturer’s instructions (Miltenyi Biotec). Cells were stimulated with plate-bound anti-CD3 (5 μg/ml) and anti-CD28 (5 μg/ml) monoclonal antibodies in the presence of different cytokines and neutralizing antibodies as specified: IL-12 (4 ng/ml; Peprotech), for Th1 differentiation; IL-4 (10 ng/ml; Peprotech) and anti-IFNγ antibody (10 μg/ml; eBioscience, clone XMG1.2) for Th2 differentiation. After 4 days, cells were expanded with fresh medium for 3 days, restimulated with plate-bound anti-CD3 (0.5 μg/ml) and anti-CD28 (0.5 μg/ml) monoclonal antibodies, and analysed by flow cytometry.

### BrdU incorporation

For *in vivo* BrdU labelling experiments, mice received two i.p. injections of BrdU (BD Biosciences; 1 mg/6 g of mouse weight) at 24 h and 12 h before sacrifice. Cells were surface stained, fixed, permeabilized and intracellularly stained using a BrdU Flow kit (BD Biosciences).

### Western blot

Cell lysates were prepared by homogenising PBS-washed cells in SDS-sample buffer (50 mM Tris-HCl (pH 6.8), 1% (v/v) β-mercaptoethanol, 2% (w/v) SDS, 0.01% (w/v) Bromophenol blue, 10% (v/v) glycerol). Proteins were resolved by SDS-PAGE, and analysed by standard western blotting techniques, using the following antibodies: PARP-1 (Bio-Rad, clone A6.4.12), in-house generated affinity purified rabbit polyclonal antibody raised against PARP-2 residues 1–69 fused to GST, β-Actin (Sigma-Aldrich, clone AC-15). Primary antibody binding was detected using peroxidase-coupled rabbit anti-mouse (Dako) or goat anti-rabbit Ig antibodies (Promega) according to the manufacturer’s protocol.

### PARP activity

Protein extracts were prepared from purified T-cells upon stimulation with anti-CD3 (5 μg/ml) and anti-CD28 (5 μg/ml) monoclonal antibodies for 14 h as previously described^60^. PARP activity was determined by using 10 μg of total protein and the HT Universal Colorimetric PARP Assay Kit with Histone-Coated Strip Wells (Trevigen, Gaithersburg, MD) following manufacturer’s instructions.

### Comet assay

Alkaline comet assay on CD4^+^ and CD8^+^ sorted cells was performed with CometAssay kit (Trevigen) following manufacturer’s instructions.

### Mixed bone marrow chimeras

Competitor bone marrow cells (1 × 10^6^) from wild-type B6.SJL mice expressing the CD45.1 leukocyte cell surface marker were mixed at a 1:1 ratio with donor bone marrow cells (1 × 10^6^) of either *Cd4-cre;Parp-2*^*f/f*^*;Parp-1*^−/−^ or control mice (*Cd4-cre;Parp-2*^+/+^*;Parp-1*^+/+^) expressing the CD45.2 marker and injected intravenously into lethally irradiated (9.5 Gy) B6 × B6.SJL F1 (CD45.1/CD45.2) recipient mice. Reconstitution was analyzed 10 weeks later.

### T-dependent antibody response

Eight-to-ten-week-old animals were immunized intraperitoneally with 100 μg/mouse of trinitro-phenyl-conjugated keyhole limpet hemocyanin (TNP-KLH) (Biosearch Technologies) in Sigma Adjuvant System (Sigma-Aldrich). Serum was collected from tail vein at 14 days after immunization. An ELISA assay was applied for quantification of TNP-specific Igs in the sera as indicated in the supplementary data.

### Peptides

Peptides OVA_257-264_ (SIINFEKL) from ovalbumin and B8R_20–27_ (TSYKFESV) from VACV soluble IFNγ receptor homologue B8R^61^ were synthesized in an Applera peptide synthesizer model 433 A, purified, and determined to be homogeneous by HPLC analysis.

### Virus assays

VACV strain WR and the recombinant virus encoding full-length OVA (rVACV-flOVA) based on the WR strain were provided by J.W. Yewdell and J. Bennink (National Institutes of Health, Bethesda, MD). Recombinant VACV expressing GFP based on the WR strain (rVACV-GFP) was provided by R. Blasco (INIA, Madrid, Spain). Stocks were grown in CV1 (African green monkey kidney cells) monolayers cultured in DMEM with 10% FBS, and consisted of clarified sonicated cell extracts. For virus titration *ex vivo*, ovaries were dissected from infected mice, mechanically homogenized and subjected to five freeze-thaw cycles. Alternatively, ovaries of infected mice were frozen at −80 °C in tubes with sterilized ceramic beads, thawed and processed using MagNA Lyser (Roche, Basel, Switzerland) at 3000 × g for 30 seconds. Ten-fold serial dilutions of each ovary homogenate were inoculated onto CV1 cells, and cells were stained 24 h later with crystal violet.

### Bone marrow–derived dendritic cells

Bone marrow–derived dendritic cells (BMDC) were generated from bone marrow progenitors^62^ as indicated in supplementary data.

### Viral infection and DC vaccination of mice

Mice were intraperitoneally infected with 1 × 10^6^ PFU of rVACV-flOVA and at 7-9 d.p.i. spleens, peritoneal exudate cells (PEC) and ovaries were obtained for further analysis. To analyze the CD8^+^ secondary response, mice were vaccinated with 1 × 10^6^ matured DC pulsed with B8R_20-27_ and OVA_257-264_ peptides, infected 9-21 days later with 1 × 10^6^ PFU of rVACV-flOVA and 5 days later, spleens, PEC and ovaries were extracted for further analysis. An ELISA assay was applied for quantification of VACV-specific antibodies, as indicated in the supplementary data.

### Histology

Tissue histology was performed as indicated in supplementary data.

### Statistical analysis

Results are presented either as mean values ± SEM or else the values for individual mice are plotted and the median value is shown. The log-rank test was used to determine the statistics of animal survival. For all other statistical analyses, either parametric or non parametric tests were applied after data were checked for normality using both D’Agostino-Pearson omnibus and Shapiro-Wilk normality tests. Data were considered to fit a normal distribution only if they passed both tests. Parametric one-way ANOVA test followed by a post-hoc Tukey test was applied. When not, ANOVA non-parametric Kruskal-Wallis test were applied to all data to determine the equality of all genotypes. When the null hypothesis that all genotypes were equal was rejected with a p-value > *, a post-hoc Dunn’s test was performed for all possible pairs of data and identified those genotypes that were significantly different, which are indicated in the figures. P values of less than 0.05 were considered to indicate statistical significance.

## Additional Information

**How to cite this article:** Navarro, J. *et al*. PARP-1/PARP-2 double deficiency in mouse T cells results in faulty immune responses and T lymphomas. *Sci. Rep.* **7**, 41962; doi: 10.1038/srep41962 (2017).

**Publisher's note:** Springer Nature remains neutral with regard to jurisdictional claims in published maps and institutional affiliations.

## Supplementary Material

Supplementary Information
