# Supplementary material for: PARP-1/PARP-2 double deficiency in mouse T cells results in faulty immune responses and T lymphomas
Source: Sci Rep. 2017 Feb 9;7:41962. doi: 10.1038/srep41962 (PMC5299517; doi:10.1038/srep41962)
Supplement: Supplementary Information [file srep41962-s1.pdf]

Supplementary data

PARP-1/PARP-2 double deficiency in mouse T-cells results in faulty immune responses and T lymphomas

Judith Navarro[1], Beatriz Gozalbo-López[2], Andrea C. Méndez[2], Françoise Dantzer[3], Valérie Schreiber[3], Carlos Martínez[4,5], David M. Arana[2], Jordi Farrés[1], Beatriz Revilla-Nuin[5,6], María F. Bueno[2], Coral Ampurdanés[1], Miguel A. Galindo-Campos[1], Philip A. Knobel[7], Sandra Segura-Bayona[7], Juan Martin-Caballero[8], Travis H. Stracker[7], Pedro Aparicio[9], Margarita Del Val[2*], José Yélamos[1,5,10*]

[1]Cancer Research Program, Hospital del Mar Medical Research Institute (IMIM), Barcelona, Spain; [2] Inmunología Viral, Centro de Biología Molecular Severo Ochoa (CSIC-UAM), Madrid, Spain; [3]Biotechnology and Cell Signaling, UMR7242-CNRS, Laboratory of Excellence Medalis, ESBS, Illkirch, France; [4] Experimental Pathology Unit, IMIB-LAIB-Arrixaca, Murcia, Spain; [5]CIBERehd, Spain; [6]Genomic Unit. IMIB-LAIB-Arrixaca, Murcia, Spain; [7]Institute for Research in Biomedicine (IRB Barcelona), The Barcelona Institute of Science and Technology, Barcelona, Spain; [8]Barcelona Biomedical Research Park (PRBB), Barcelona, Spain; [9]Department of Biochemistry, Molecular Biology and Immunology, University of Murcia, Murcia, Spain; [10]Department of Immunology, Hospital del Mar, Barcelona, Spain.

Generation of *Parp-2$^{flox/flox}$* mice

For the establishment of the *Parp-2$^{flox/flox}$* (*Parp-2$^{f/f}$*) mouse a targeting vector was constructed as follows. A 0.34 kb fragment encompassing exon 8 was amplified by PCR (from 129S2/SvPas ES cells genomic DNA) and subcloned in an MCI proprietary vector. This MCI vector contains a LoxP site as well as a floxed and flipped Neomycin resistance cassette. A 3 kb fragment (corresponding to the 5' homology arm) and 2.87 kb fragment (corresponding to the 3' homology arms) were amplified by PCR and subcloned in step1 plasmid to generate the final targeting construct. The linearized

construct was electroporated in 129S2/SvPas mouse embryonic stem (ES) cells. After selection, targeted clones were identified by PCR using external primers and further confirmed by Southern blot with 5' and 3' external probes. Two positive ES clones were injected into C57BL/6J blastocysts, and male chimaeras derived gave germline transmission.

Flow cytometry antibodies

The following antibodies were used in flow cytometry analyses:

FITC-, PerCP-, and APC-conjugated anti-CD8 (BioLegend, clone 53-6.7 and Proimmune, clone KT15), PECy7- and APC-conjugated anti-CD4 (BioLegend and Immunotools, respectively, clone GK1.5), FITC-conjugated anti-CD24 (BD Biosciences, clone M1/69), PE-conjugated anti-TCRβ (BD Biosciences, clone H57-597), APC-conjugated anti-CD127 (IL7Rα) (BioLegend, clone A7R34), FITC- and PerCP-Cy5.5-conjugated anti-CD44 (Immunotools and BD Biosciences, clone IM7), FITC-, PE- and APC-conjugated anti-CD62L (eBioscience and Immunotools, clone MEL-14), PE-conjugated anti-IFNγ (Biolegend, clone XMG1.2), FITC-conjugated anti-IL2 (BD Pharmingen, clone JE56-5H4), PE-conjugated anti-GranzymeB (Invitrogen, clone GB12), PerCP-conjugated anti-B220 (CD45R) (BD Biosciences, clone RA3-6B2), APC-Cy7-conjugated anti-CD3 (BD Biosciences, clone 17A2), FITC-conjugated anti-CXCR5 (BD Biosciences, clone 2G8), PE-conjugated anti-CD278 (ICOS) (BioLegend, clone 7E.17G9), APC-conjugated anti-CD279 (PD-1) (BD Biosciences, clone J43), PerCP-conjugated and biotinylated anti-CD69 (BioLegend, clon H1.2F3), APC-Cy7-conjugated anti-CD45.1 (BD Biosciences, clone A20), FITC-conjugated anti-CD45.2 (BD Biosciences, clone 104), FITC-conjugated anti-active-caspase-3 (BD Biosciences, clone C92-605), FITC-conjugated anti-γH2AX (Ser139) (Millipore, clone

JBW301), APC-conjugated anti-CD11c (eBioscience, clone N418), PE-conjugated anti-CD86 (Biolegend, clone GL-1), PE-conjugated anti-CD80 (BD Pharmingen, clone 16-10A1), FITC-conjugated anti-H-2K^b (BD Pharmingen, clone AF6-88.5), FITC-conjugated anti-I-A^b (BD Pharmingen, clone AF6-120.1), APC-conjugated anti-CD25 (eBioscience, clone PC 61.5); PE-conjugated anti-FoxP3 (eBioscience, clone FJK-16s), APC-conjugated anti-IFNγ (eBioscience, clone XMG1.2), APC-conjugated anti-IL-4 (eBioscience, clone 11B11) as well as FITC- and Alexa647-labeled streptavidin (Beckman Coulter).

ELISA

TNP-specific Igs in the sera of TNP-KLH immunized mice were evaluated by ELISA. Ninety-six-well plates were coated with TNP-BSA (Biosearch Technologies) in PBS/Tris-HCl 0.1M and incubated overnight at 4ºC. After blocking with PBS and 1% BSA, dilutions of mouse serum were added, and again plates were incubated overnight at 4ºC. The following day biotinylated IgM (clone R6-60.2), IgG1 (clone A85-1), IgG2a (clone R19-15), IgG2b (clone R12-3), and IgG3 (clone R40-82) antibodies (BD Biosciences) were added and plates were incubated for 1 h at room temperature. After washing, the plates were incubated with streptavidin-horseradish peroxidase (Dako) for 1 h at room temperature. Following washing, 3,3′,5,5′-Tetramethylbenzidine (Sigma-Aldrich) was added and the plates were read at 690 nm.

Anti-VACV humoral response

For quantification of VACV-specific antibodies, ninety-six-well plates were coated with 1 μg/well of protein extract from BSC-1 cells infected with VACV-WR [1]. Antibodies were detected in heat-inactivated serum from infected mice using an anti-Ig (G+M)-

HRP antibody and adding OPD (Dako) as substrate. Optical density was measured at 450 nm, and antibody units (AbU) per mL were calculated as described [2]. All serum samples were analyzed in three independent dilutions, each one in duplicate. Neutralizing antibodies were measured by plaque reduction, incubating serial dilutions of heat-inactivated serum from experimental infected mice with 200 plaque forming units of VACV-WR for 1 h at 37ºC. These serum-virus mixtures were inoculated onto CV1 cells, and stained 24 h later for plaque formation. Neutralization titer (NT50) was defined as the serum dilution which reduces 50% the viral titer obtained in the presence of non-immune serum. Each serum sample was analyzed in triplicate.

Immunohistology

Tissue samples were collected and fixed in 4% buffered-formalin for 36 hours, processed and paraffin-embedded. Three micrometers-wide sections were then obtained and stained with a standard hematoxylin and eosin for histopathological examination. For immunohistopathologic characterization of lymphoid tumours, an indirect ABC immunohistochemical procedure was performed using a polyclonal rabbit anti-CD3 antigen (Dako) for T-cell characterization. For evaluation of the infiltrative degree of tumors, a semi-quantitative scale comprised 4 grades was used: 0 (no infiltration), 1 (mild infiltration), 2 (moderate infiltration) and 3 (severe infiltration). The overall infiltrative value of each organ was established by calculation of the mean of all values obtained.

Bone marrow–derived dendritic cells

Bone marrow–derived dendritic cells (BMDC) were generated from bone marrow progenitors in the presence of GM-CSF-containing supernatant obtained from J558 cells grown in RPMI (Lonza) with 10% FBS. After 7-9 days, nonadherent cells with a typical

DC morphology and a myeloid DC phenotype (MHC class II+, CD11c+, and CD8-) were collected. For ex vivo assays, immature DC were infected overnight with rVACV-flOVA at a multiplicity of infection (MOI) of 5. For vaccination, DC were incubated with peptides $B8R_{20-27}$ or $OVA_{257-264}$, matured with 1 μg/ml LPS for 1 h. For phenotypical studies, immature DC were used. Susceptibility to infection to VACV was assessed by infecting DC with rVACV-GFP and staining for CD11c expression. Naïve T-lymphocyte priming assays were performed infecting DC with rVACV-flOVA. Infected BMDCs were then co-cultured with splenocytes from OT-I TCR transgenic naïve mice for 1h and then for 4h more in the presence of brefeldin A (Sigma). Cells were then surface stained with anti-CD8 antibody and intracellularly stained with anti-IFNγ.

Supplementarty references

1. Gomez,C.E. *et al.* Head-to-head comparison on the immunogenicity of two HIV/AIDS vaccine candidates based on the attenuated poxvirus strains MVA and NYVAC co-expressing in a single locus the HIV-1BX08 gp120 and HIV-1(IIIB) Gag-Pol-Nef proteins of clade B. *Vaccine* **25**, 2863-2885 (2007).
2. Miura,K. *et al.* Development and characterization of a standardized ELISA including a reference serum on each plate to detect antibodies induced by experimental malaria vaccines. *Vaccine* **26**, 193-200 (2008).

Supplementary Figure legends

Figure S1.- Treg cells compartment in mice with a T-cell-specific deletion of PARP-2 in a PARP-1-deficient background. (A) Representative dot-plots of CD25, and FoxP3 expression in $CD4^+$-gated splenocytes of the indicated genotypes. Percentage of cells in the individual subpopulations is indicated in each quadrant. (B) Graph showing the percentage of Treg cells ($CD4^+CD25^+FoxP3^+$). Values represent the mean ± SEM of at least 9 mice of each genotype. *, $P<0.05$.

Figure S2.- PARP-1/PARP-2 doubly-deficient T-cells had an intrinsic defect. Competitor bone marrow cells from wild-type B6.SJL mice expressing the CD45.1 leukocyte cell surface marker were mixed at a 1:1 ratio with donor bone marrow cells of either *Cd4-cre;Parp-2*$^{f/f}$*;Parp-1*$^{-/-}$ or control mice (*Cd4-cre;Parp-2*$^{+/+}$*;Parp-1*$^{+/+}$) expressing the CD45.2 marker and injected intravenously into lethally irradiated (9.5 Gy) B6 x B6.SJL F1 (CD45.1/CD45.2) recipient mice. Reconstitution was analyzed 10 weeks later. Representative dot-plots of CD45.1^{+} and CD45.2^{+}-expressing cells in naïve and memory CD4^{+} and CD8^{+} T-cells from spleen. Percentage of cells in the individual subpopulations is indicated in each quadrant.

Figure S3.- IL-7 response does not account for the T-cell defect in PARP-1/PARP-2 doubly deficient T cells. Representative histograms and quantification of IL-7Rα protein expression by flow cytometry in CD4SP, and CD8SP thymocytes (A) and in CD4^{+}CD62L^{+}CD44^{lo} and CD8^{+}CD62L^{+}CD44^{lo} splenocytes (B) derived from *Cd4-cre;Parp-2*$^{+/+}$*;Parp-1*$^{+/+}$, *Cd4-cre;Parp-2*$^{f/f}$*;Parp-1*$^{+/+}$, *Cd4-cre;Parp-2*$^{+/+}$*;Parp-1*$^{-/-}$, and *Cd4-cre;Parp-2*$^{f/f}$*;Parp-1*$^{-/-}$ mice. MFI, mean fluorescent intensity. Thymocytes (C) and splenocytes (D) from *Cd4-cre;Parp-2*$^{+/+}$*;Parp-1*$^{+/+}$, *Cd4-cre;Parp-2*$^{f/f}$*;Parp-1*$^{+/+}$, *Cd4-cre;Parp-2*$^{+/+}$*;Parp-1*$^{-/-}$, and *Cd4-cre;Parp-2*$^{f/f}$*;Parp-1*$^{-/-}$ mice were cultured in medium supplemented or not with 10 ng/ml recombinant IL-7 (rIL7) for different times. The proportion of live (annexinV^{-}Propidium iodide^{-}) cells was assessed by flow cytometry. **, P<0.01; ***, P<0.001.

Figure S4.- **Efficient class switch recombination in B cells from mice with a T-cell-specific deletion of PARP-2 in a PARP-1-deficient background.** Graph showing the surface expression of IgG1, IgG2a, IgG2b and IgG3 analyzed by flow cytometry in B

lymphocytes of the indicated genotypes stimulated for 72 h with either LPS (CSR to IgG2b and IgG3), or LPS + IL-4 (CSR to IgG1) or LPS + IFNγ (CSR to IgG2a). Values represent the mean ± SEM of 3 independent experiments.

Figure S5.- Primary cellular immune response in splenocytes following vaccinia virus infection. Mice of the indicated genotypes were infected with VACV and 7-9 days later spleens were extracted. (A) Flow cytometry analysis of activation molecules (CD44, CD69, CD62L) and effector molecules (IL2, IFNγ) (alone or combined) in the $CD4^+$ cell population following *ex vivo* incubation with VACV-infected dendritic cells. (B) Analysis of activation molecules (CD44, CD69, CD62L) and effector molecules (GZMB, IL2, IFNγ) (alone or combined) in $CD8^+$ cells following *ex vivo* incubation with $B8R_{20\text{-}27}$ peptide. Dots represent individual mice and horizontal lines represent median values of cell % for each genotype of the same experiments for which absolute cell numbers are plotted in Figure 6. *, $P<0.05$; **, $P<0.01$; ***, $P<0.001$.

Figure S6.- *In vitro* T-cell cytokine production. Representative dot-plots of IFNγ (A) and IL-4 (B) expression in naïve $CD4^+$ T-cells from mice of the indicated genotypes differentiated *in vitro* in Th1 (A) or Th2 (B) conditions. Percentage of cells in the individual subpopulations is indicated in each quadrant. Graph showing the percentage of $IFN\gamma^+$ (C) and $IL\text{-}4^+$ (D) cells. Data are from two independent experiments and values represent the mean ± SEM. *, $P<0.05$; ***, $P<0.001$.

Figure S7.- Primary cellular immune response in peritoneal exudate cells following vaccinia virus infection. Mice of the indicated genotypes were infected with VACV and 7-9 days later peritoneal cells were lavaged. Flow cytometry analysis of activation

molecules (CD44, CD69, CD62L) and effector molecules (IL2, IFNγ) (alone or combined) following incubation with VACV-infected DCs in $CD4^+$ cells (A) or with $B8R_{20-27}$ peptide in $CD8^+$ cells (B). Dots represent individual mice and horizontal lines represent median values for each genotype. *, P<0.05; **, P<0.01.

Figure S8.- BMDC maturation and function. Cells were extracted from bone marrow of mice of the genotypes indicated and differentiated to BMDCs in culture the presence of GM-CSF for 7-9 days. (A) Differentiation to BMDCs was measured by CD11c expression by flow cytometry. Maturation was studied measuring MHC class I $H\text{-}2K^b$, CD80, and MHC class II $I\text{-}A^b$ in $CD11c^+$ cells and CD86 in $CD11c^+CD86^{hi}$ cells (n=2-6 experiments). gMFI, geometric mean fluorescence intensity. (B) Susceptibility to infection by VACV was measured infecting BMDCs with rVACV-GFP *ex vivo* for 5 h and quantitating $CD11c^+$ cells expressing GFP (n=4, one representative experiment is shown). (C) T-lymphocyte priming assays: BMDCs infected with rVACV-flOVA with a multiplicity of infection (MOI) of 6 were co-cultured with splenocytes from naïve OT-I TCR transgenic mice. Activation of $CD8^+$ T cells was detected by the intracellular expression of IFNγ by flow cytometry (one representative experiment out of four is shown).

Table S1.- List of oligonucleotides

Oligo Name	Oligo sequence (5' to 3')
Parp-2$^{f/f}$-forward (A)	CTCGAGTGTTTCACTGTGAGGGAG
Parp-2$^{f/f}$-reverse (B)	CCCCAAACCAGAGTCCCATCC
Cd4-cre-forward	TCGATGCAACGAGTGATGAGGTTCG
Cd4-cre-reverse	ACAGCATTGCTGTCACTTGGTCGTG
Parp-1 (+)	GGCCAGATGCGCCTGTCCAAGAAG
Parp-1 (+/-)	CTTGATGGCCGGGAGCTGCTTCTTC
Parp-1 (-)	GGCGAGGATCTCGTCGTGACCCATG

A

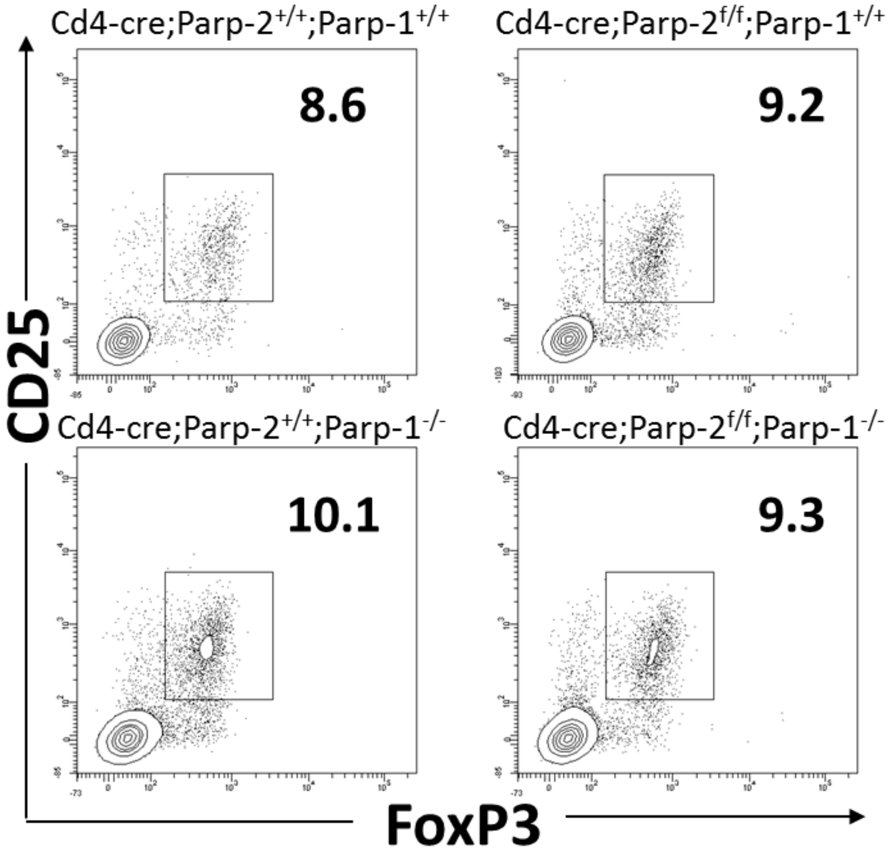

B

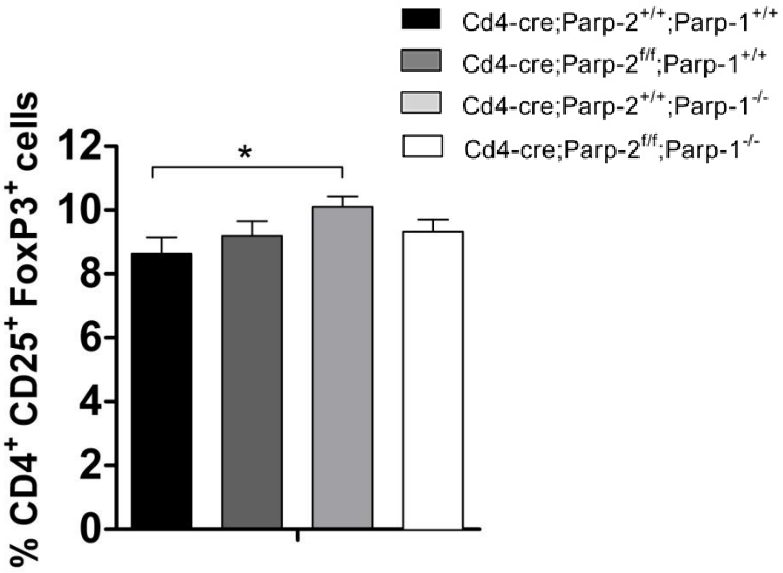

Navarro et al., Fig. S1

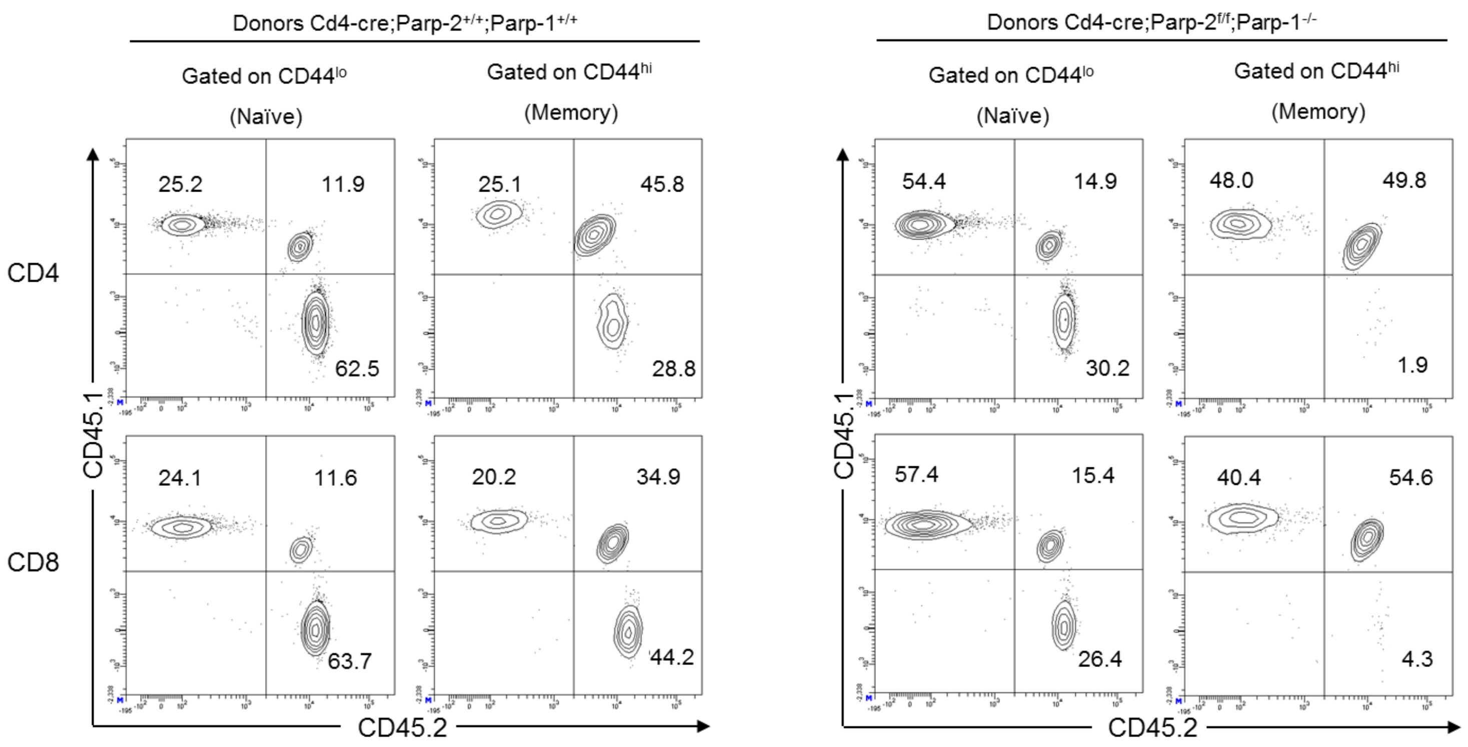

Navarro et al., Fig. S2

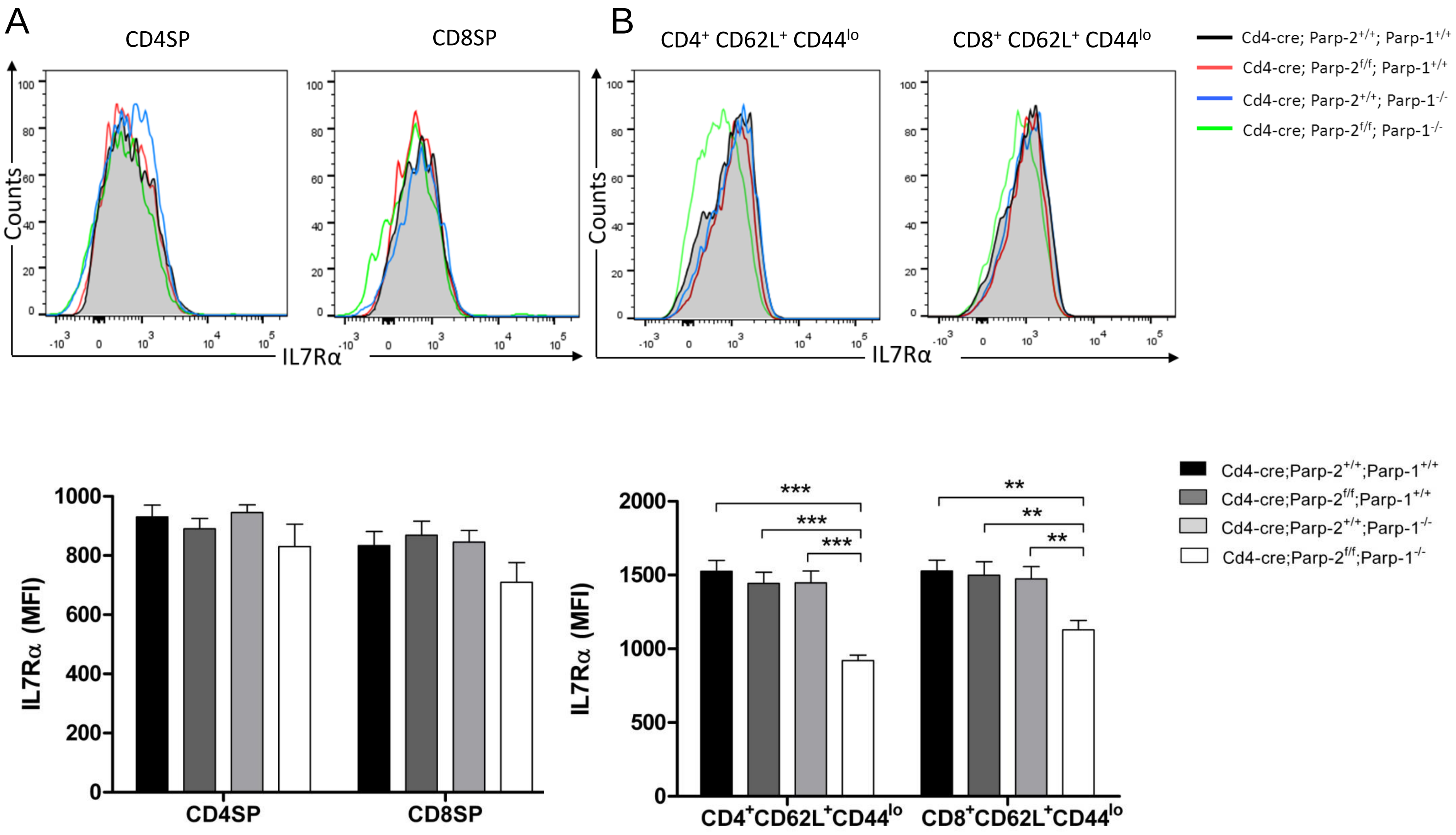

Navarro et al., Fig. S3

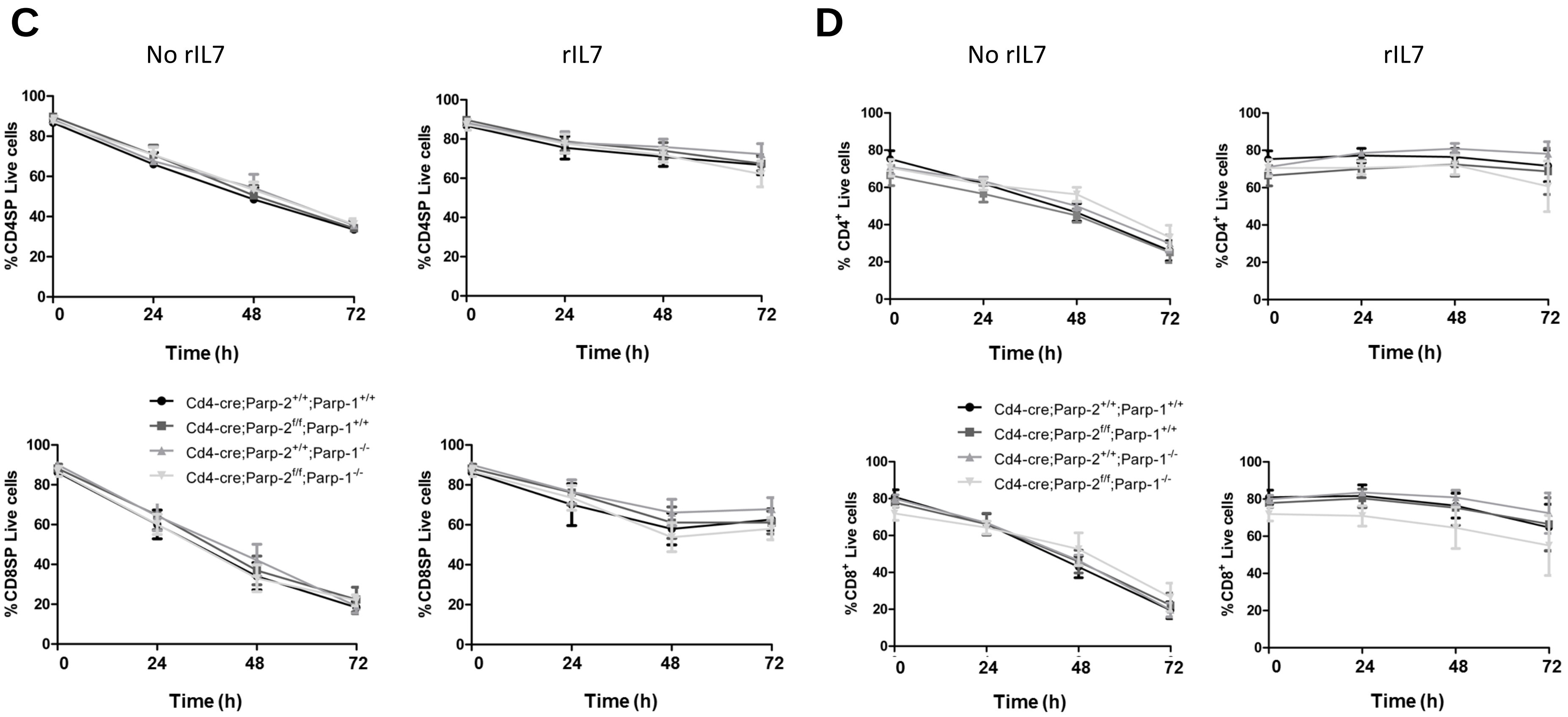

Navarro et al., Fig. S3

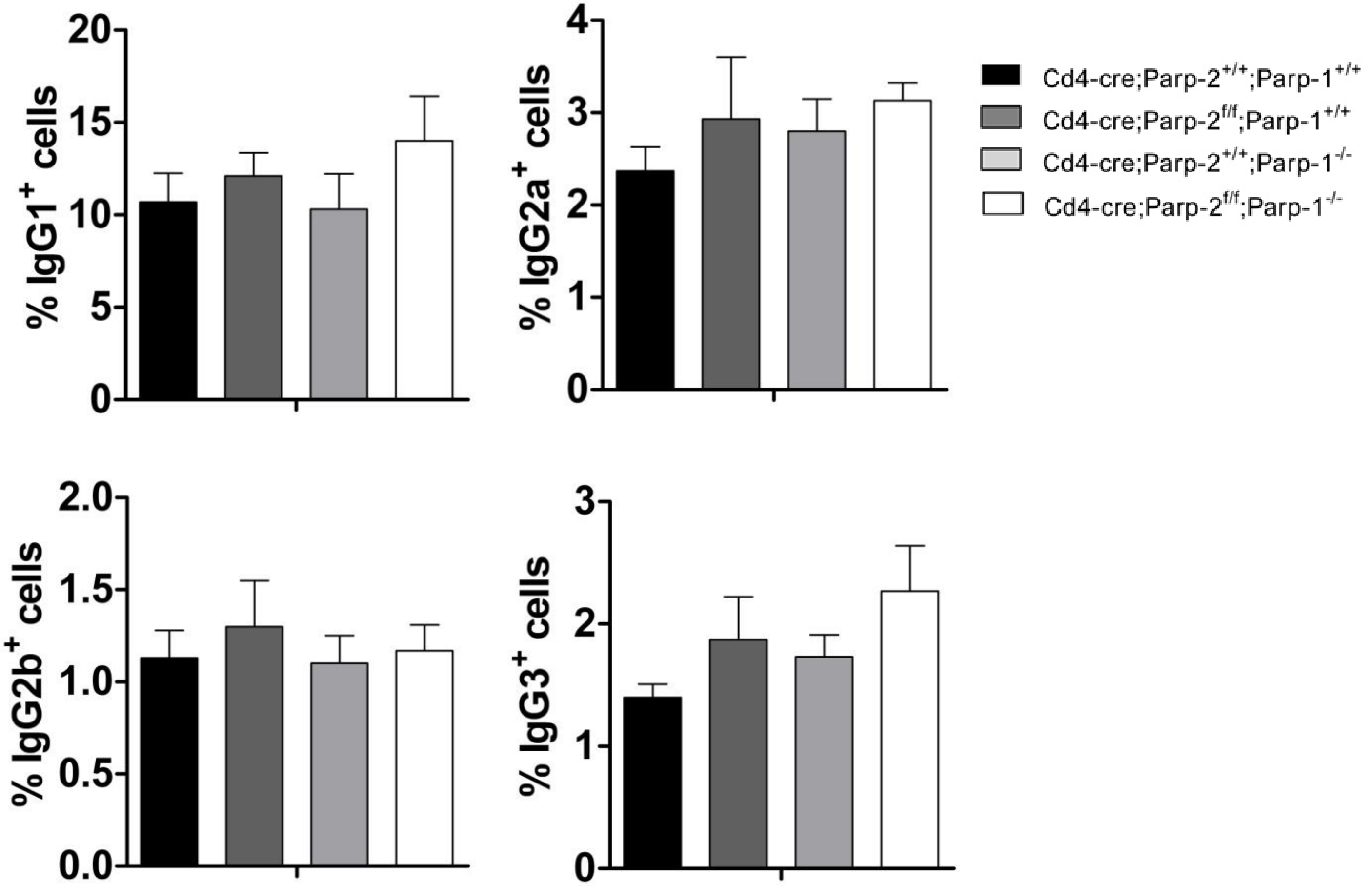

Navarro et al., Fig. S4

Primary infection, spleen, %

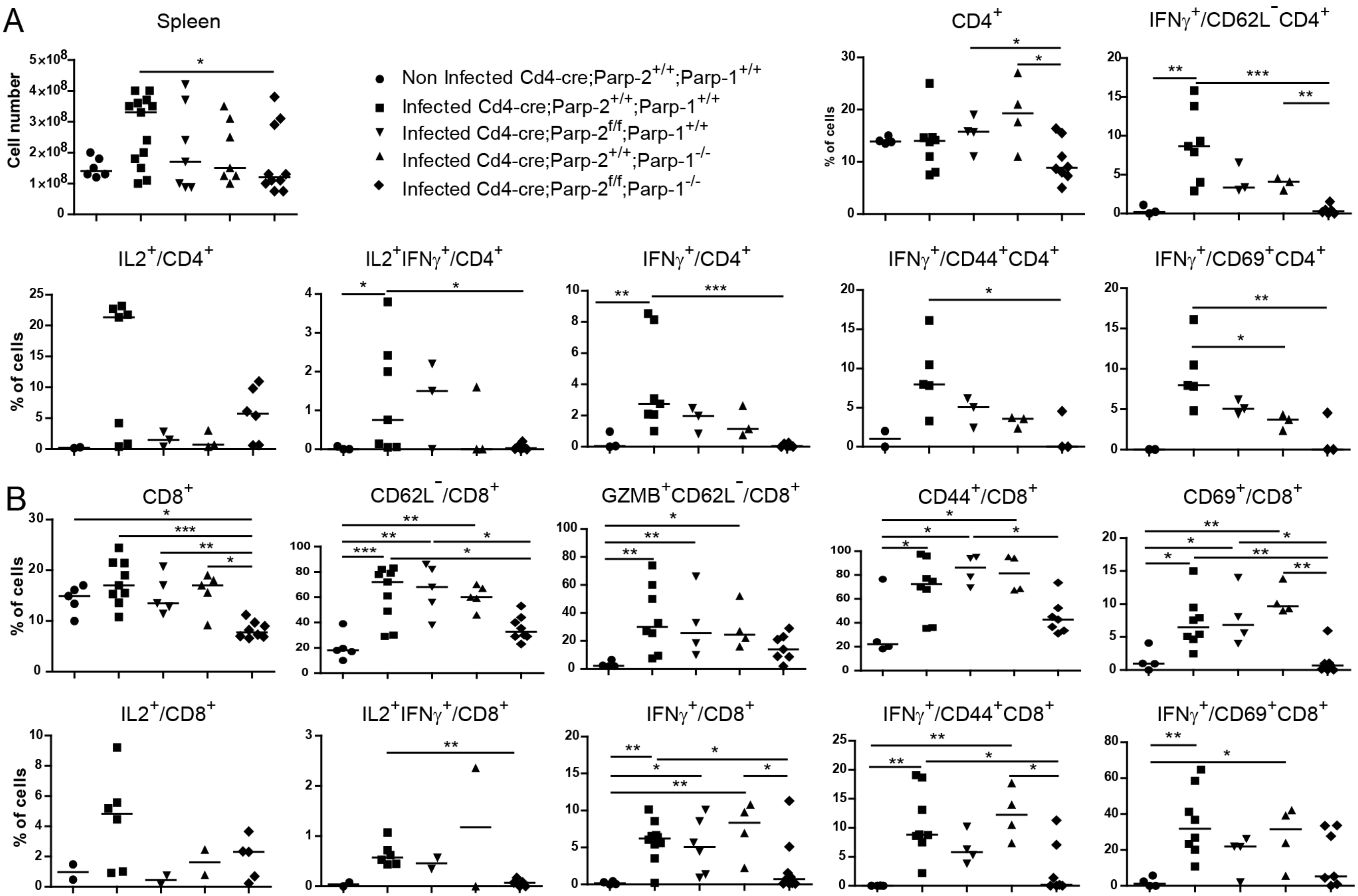

Navarro et al., Fig. S5

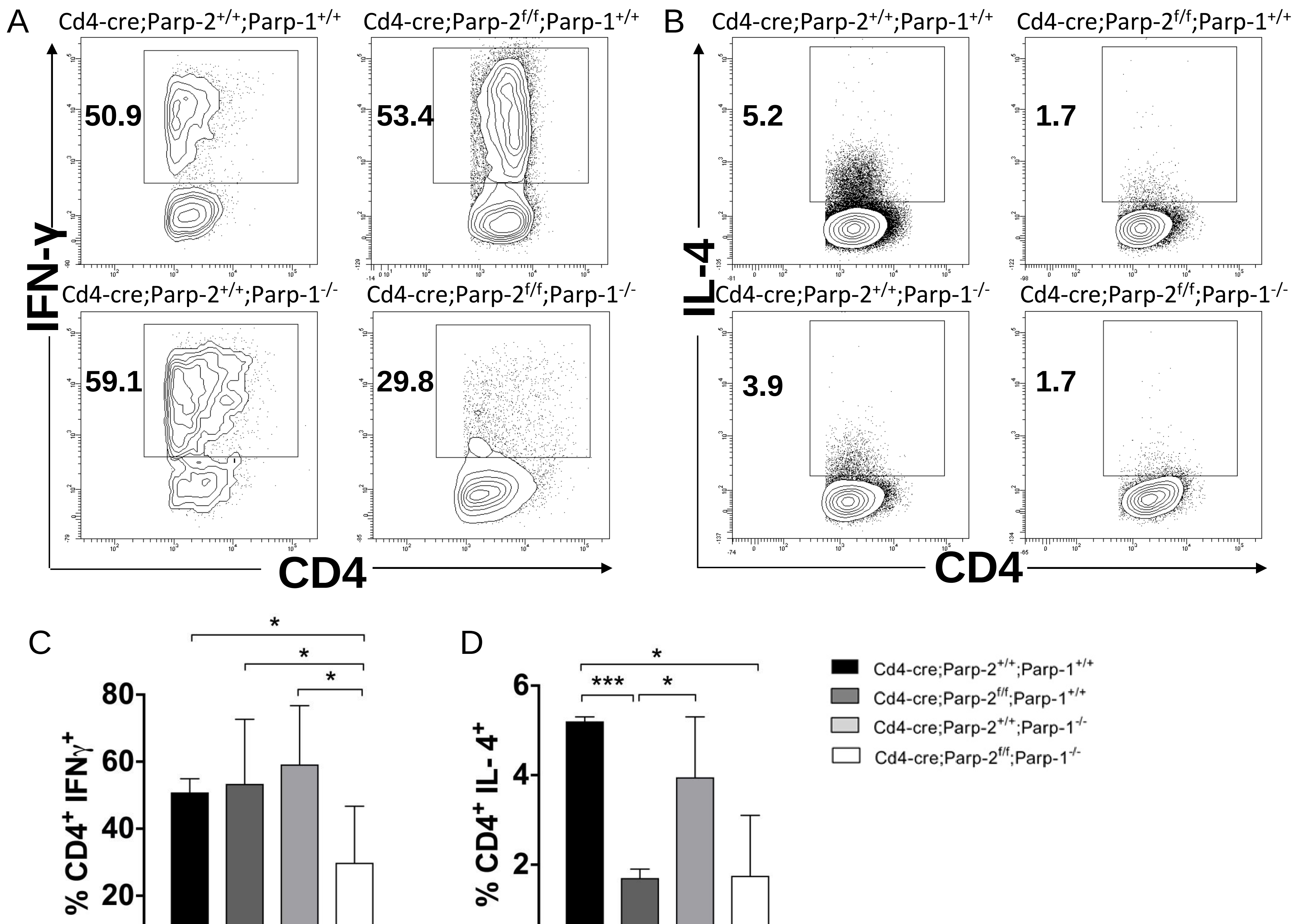

Navarro et al., Fig. S6

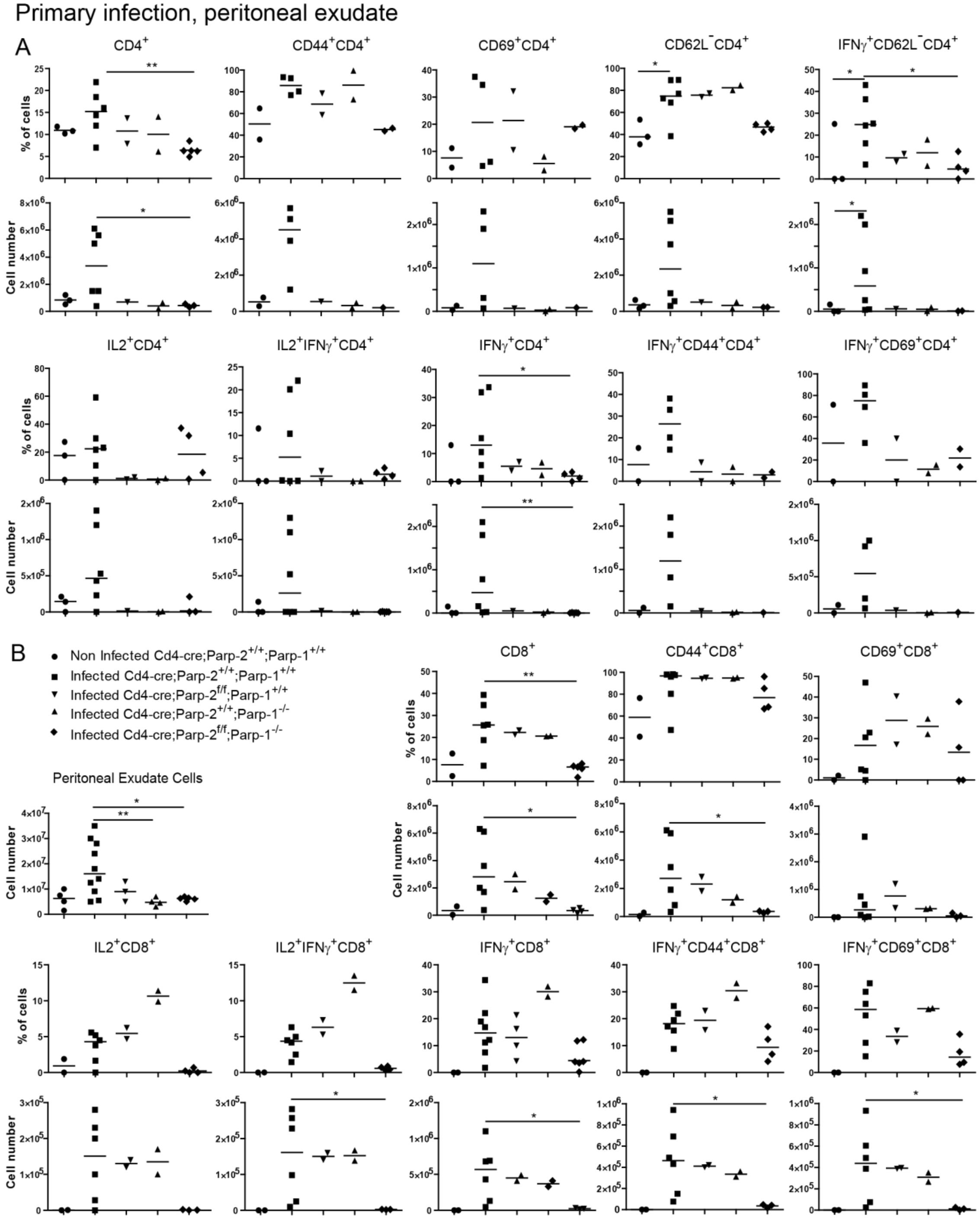

Navarro et al., Fig. S7

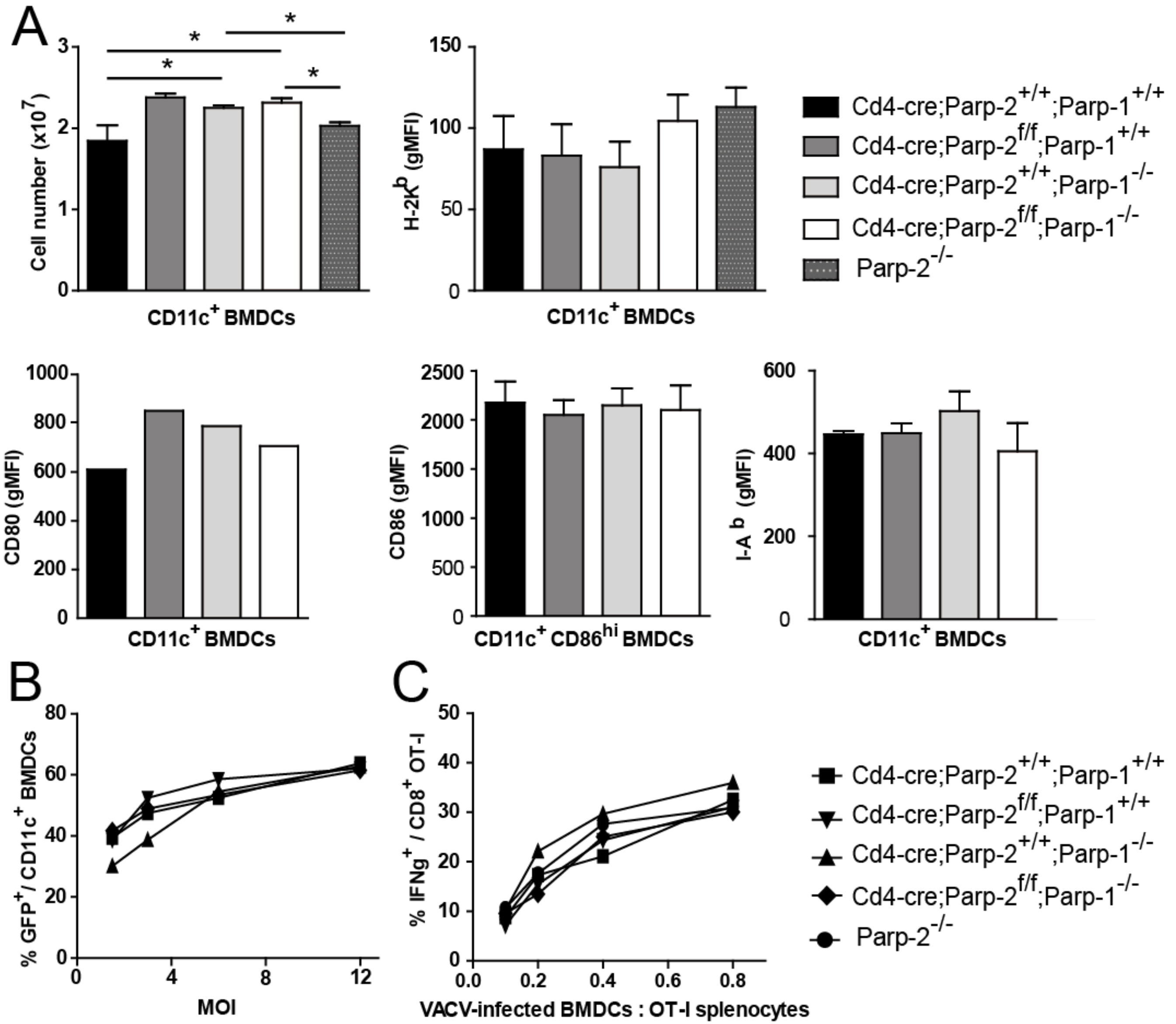

Navarro et al., Fig. S8
